# Gut Microbiomes of Rainbow Trout and Atlantic Salmon: Nutritional Modulation, Mucosal Immunity, and Resistome Risk

**DOI:** 10.3390/biology15131066

**Published:** 2026-07-03

**Authors:** Zhongquan Jiang, Jiale Chen, Yuanhao Ren, Tingting Lin, Siping Li, Fengyuan Shen, Bo Qin, Lei Li, Changjian Li, Na Ying, Hanfeng Zheng

**Affiliations:** 1 Key Laboratory of East China Sea Fishery Resources Exploitation, Ministry of Agriculture and Rural Affairs, P.R. China, East China Sea Fisheries Research Institute, Chinese Academy of Fishery Sciences, Shanghai 200090, China; zhongquanj@sjtu.edu.cn (Z.J.); renyh@ecsf.ac.cn (Y.R.); lintt@ecsf.ac.cn (T.L.); lisiping@ecsf.ac.cn (S.L.); fyshen0817@126.com (F.S.); dhsqb1026@126.com (B.Q.); zheyilee@126.com (L.L.); xyz6622@126.com (N.Y.); 2 Jiangsu Marine Salmon & Trout Science and Technology Innovation Center, Yancheng 224500, China; 3 Key Laboratory of Environmental Health Impact Assessment of Emerging Contaminants, Ministry of Ecology and Environment, School of Environmental Science and Engineering, Shanghai Jiao Tong University, Shanghai 200240, China; jialechen@njau.edu.cn; 4 Lianyungang Ocean and Fishery Development Promotion Center, Lianyungang 222000, China; xyz66221@126.com

**Keywords:** rainbow trout, Atlantic salmon, gut microbiome, nutritional modulation, mucosal immunity, aquaculture stress, antibiotic resistance genes, resistome, multi-omics, precision aquaculture

## Abstract

The gut microbiome of rainbow trout (*Oncorhynchus mykiss*) and Atlantic salmon (*Salmo salar*) is increasingly recognized as a functional interface linking dietary inputs, epithelial barrier integrity, mucosal immunity, environmental stress, disease susceptibility, and antimicrobial-resistance risk in intensive aquaculture. Based on available salmonid studies and relevant evidence from broader fish and aquaculture systems, this review synthesizes current knowledge on salmonid gut microbial composition, nutritional modulation, microbiome–mucosal immune interactions, aquaculture stressors, antibiotic exposure, antibiotic resistance genes (ARGs), mobile genetic elements (MGEs), metagenomics, multi-omics, and emerging microbiome-informed decision-support tools. Current evidence does not support a universally stable single-core microbiota in these species. Instead, community structure is shaped by developmental stage, freshwater–seawater transition, intestinal segment, digesta versus mucosa sampling, diet, temperature, stress, health status, and methodological workflow. Feed substitution and functional additives can remodel the gut microbiota, but these shifts should be interpreted alongside histology, barrier function, metabolic profiles, immune indicators, and disease-resistance phenotypes. Antibiotic exposure may reduce acute bacterial disease pressure while disturbing community structure and potentially enriching ARGs or ARG–MGE associations. Risk assessment should therefore move beyond ARG abundance toward host–ARG–MGE linkage using shotgun metagenomics, metagenome-assembled genomes, long-read sequencing, Hi-C, and externally validated multi-omics models. Machine learning and artificial intelligence approaches may support feature screening, risk stratification, and decision support, but their application in salmonid gut-health management remains at an early stage and requires external validation across sites, production stages, diets, and seasons.

## Simple Summary

Rainbow trout and Atlantic salmon are major cold-water farmed fish, and their intestinal microbes are increasingly viewed as part of gut health and production resilience. These microbial communities respond to feed ingredients, temperature, seawater transfer, handling, disease pressure, and antibiotic treatment, while also interacting with the mucus layer, intestinal barrier, and immune system. This review explains how diet replacement and functional feed additives can reshape the gut microbiome of these salmonids, why microbial change alone should not be interpreted as either beneficial or harmful, and how antibiotic use may affect resistance genes in fish and the surrounding farm environment. We emphasize that microbiome results need to be interpreted together with intestinal structure, barrier function, immune responses, metabolites, and disease outcomes. Future progress will require standardized sampling, long-term monitoring, shotgun metagenomics, multi-omics integration, and carefully validated analytical models. Such approaches can support more sustainable salmonid farming, better gut-health management, and more responsible antimicrobial use.

## 1. Introduction

### 1.1. Fundamental Biological Functions

The fish gut microbiome is increasingly recognized as more than a passive component of the digestive tract; it represents a key host-associated interface linking feed digestion, nutrient absorption, epithelial renewal, immune homeostasis, and pathogen defense [1,2,3,4,5]. Studies in gnotobiotic zebrafish have shown that gut microbial colonization induces host transcriptional responses that are relatively conserved with those in mammals, shifting fish gut microbiota research from community description toward mechanisms of host regulation [1]. Subsequent in vivo fluorescent fatty acid tracing further demonstrated that the microbiota can promote fatty acid uptake by the fish intestinal epithelium and enhance lipid droplet formation in the intestine and liver, indicating direct involvement in energy acquisition and lipid metabolism [2]. At the mucosal immune interface, the discovery of IgT in rainbow trout and its preferential coating of intestinal parasites and commensal bacteria indicate that teleosts have evolved an antibody system closely associated with mucosal surfaces; the gut microbiome is therefore an endogenous component of immune development and homeostatic maintenance in fish [3,4]. Zebrafish models also show that intestinal microbes can regulate innate immune set points through Myd88-related signaling. Thus, the fish gut microbiota affects not only community composition but also the way in which the host responds to microbial signals [5]. Gnotobiotic rainbow trout models and recolonization experiments further demonstrate that endogenous bacterial communities can increase host resistance to *Flavobacterium columnare* infection, providing relatively direct causal evidence that the fish gut microbiome contributes to pathogen interception and barrier maintenance [6]. Accordingly, in fish, especially under high-density aquaculture, the gut microbiome should be regarded as a functional layer in nutrient digestion, energy metabolism, immune maturation, barrier maintenance, and pathogen defense, rather than merely an accompanying biomarker.

### 1.2. Specific Features of Salmonid Aquaculture

From an aquaculture perspective, rainbow trout and Atlantic salmon are two of the best-studied cold-water salmonids and are characterized by complex management variables, making them representative systems for examining linked “nutrition-microbiome-immunity-health” effects [7,8]. This species-specific focus is important because rainbow trout and Atlantic salmon are not simply interchangeable examples of fish gut microbiome research. These two salmonids combine high commercial value, cold-water physiology, intensive formulated-feed dependence, sensitivity to temperature and oxygen fluctuations, recurrent bacterial disease pressure, and increasing demands for antibiotic reduction, making them suitable focal taxa for linking nutrition, mucosal immunity, disease risk, and resistome monitoring within the same production-relevant framework. This relevance is also reinforced by several current production challenges in intensive salmonid aquaculture. Feed remains a major cost driver, and the replacement of fishmeal and fish oil with plant proteins, insect proteins, single-cell proteins, and other alternative ingredients can affect not only nutrient utilization but also intestinal microbial ecology, barrier status, and inflammatory responses. In Atlantic salmon, freshwater-to-seawater transfer represents a particularly vulnerable production window, during which osmoregulatory remodeling, handling stress, microbial restructuring, and post-transfer mortality can converge. In both rainbow trout and Atlantic salmon, recurrent bacterial disease and increasing pressure to reduce antibiotic use further highlight the need for gut-health indicators that can support feed evaluation, disease prevention, and antimicrobial stewardship. The continued expansion and intensification of global aquaculture have brought high-density rearing, pathogen control, ingredient replacement, and environmental stress into the same production systems, while salmonids are particularly sensitive to low temperature requirements, high dissolved oxygen demand, and water-quality fluctuations [7,8,9]. In nutritional management, Atlantic salmon is among the principal species used to study alternative protein ingredients. Both plant-protein replacement and insect-meal diets can alter gut microbiota composition, suggesting that feed substitution is not solely a formulation issue but also involves ecological restructuring of host–microbe interactions [10,11]. In environmental management, warming can markedly alter gut microbial diversity and dominant taxa in rainbow trout, whereas Atlantic salmon undergoes substantial community remodeling and short-term diversity loss after transfer from freshwater RAS production to seawater. These observations indicate that temperature shifts, water-body transitions, and transport stress may act first on the gut microecology [9,12]. In disease control, a global review of bacterial disease outbreaks in rainbow trout from 2010 to 2022 suggests that pathogen pressure remains a long-term production constraint. The gut microbiome is therefore linked simultaneously to growth, feed utilization, disease susceptibility, and health-management costs [8]. When risks of pathogen outbreaks, environmental fluctuations, and intervention measures are superimposed, the salmonid gut microbiome becomes a key interface for understanding differential host responses, evaluating the consequences of antibiotic use, and identifying risks of ARG dissemination [8,9,13,14].

### 1.3. Scope of This Review

Against this background, this review does not treat the gut microbiome of rainbow trout and Atlantic salmon merely as an object of “community compositional change”. Several broad reviews have already summarized host–microbiome interactions, diet-driven microbial modulation, disease mitigation, bioactive compounds, and aquaculture applications across fish and shellfish as a whole. We therefore do not claim that these general themes are newly identified here. Rather, the purpose of this review is to reassess these themes in a salmonid-specific context, where freshwater–seawater transition, smoltification, cold-water physiology, feed replacement strategies, mucosal immune features, and antimicrobial-management pressures create a distinct interpretive framework for rainbow trout and Atlantic salmon. Instead, it places the microbiome within an analytical framework that couples nutritional modulation, mucosal immunity, aquaculture stress, antibiotic use, and One Health risk. Although previous salmonid microbiome reviews have summarized intestinal bacterial composition, dietary modulation, and probiotic or prebiotic interventions, several important knowledge gaps remain unresolved. First, it is still unclear when diet-, development-, or stress-induced microbial shifts represent functional adaptation, transient compositional variation, or dysbiosis with consequences for barrier integrity, metabolism, and disease resistance. Second, the mechanistic links between gut microbiota and mucosal immunity, including IgT-associated bacterial coating, mucus-layer function, epithelial inflammation, and host selection of commensals versus opportunistic pathogens, remain fragmented across species, intestinal compartments, and experimental designs. Third, resistome studies in salmonid aquaculture often report ARG abundance but less frequently resolve ARG bacterial hosts, mobile genetic elements, antibiotic-exposure history, farm effluents, and One Health dissemination routes, limiting ecological risk interpretation. Fourth, microbiome-informed aquaculture management remains constrained by the lack of longitudinal, cross-site, and externally validated indicators that can be used for feed evaluation, stress monitoring, disease-risk prediction, and antimicrobial stewardship. Current research on salmonid gut microbiomes is still dominated by 16S rRNA amplicon sequencing, but meta-analytical evidence indicates that technical factors such as amplified region, DNA extraction method, and sample type can themselves significantly affect β-diversity outcomes. Therefore, cross-study comparisons of “core microbiota”, “health-associated marker taxa”, and “dysbiosis patterns” still require cautious interpretation [7]. This caution applies not only to baseline composition but also to the interpretation of diet effects, mucosal immunity, stress responses, enteritis, antibiotic exposure, and resistome risk. In particular, digesta and mucosa-associated samples do not represent the same ecological compartment. Digesta samples may more strongly capture recent feed intake, transient microbes, feed-derived microbial DNA, and luminal substrates, whereas mucosa-associated samples are more closely linked to bacterial adhesion, mucus-layer interactions, epithelial contact, and local immune selection. Differences in intestinal segment, fasting duration, time after feeding, DNA-extraction procedure, amplified 16S region, sequencing depth, reference database, and bioinformatic workflow may therefore generate apparent inconsistencies among studies even when the underlying biological processes are compatible. This also means that salmonid gut microbiome research should move beyond asking “which taxa increase or decrease” toward integrated analyses of function, mechanism, and risk.

Following this rationale, the review focuses on four interrelated questions. First, as feed formulations shift from fishmeal and fish oil toward plant proteins, insect proteins, single-cell proteins, and functional additives, do the resulting changes in the gut microbiome of rainbow trout and Atlantic salmon represent plastic adaptation, or might they progress into dysbiotic processes affecting intestinal homeostasis and health outcomes? Second, how does mucosal immunity, particularly IgT-associated bacterial coating, selection, and immune constraint, contribute to maintaining the dynamic boundary among beneficial commensals, opportunistic pathogens, and exogenous pathogens? Third, how do multiple stressors, including temperature change, water-body transition, high stocking density, transport stress, and antimicrobial intervention, jointly shape the salmonid gut microbiome–resistome and further influence disease-outbreak risk, ARG enrichment, and One Health-related environmental risk. Fourth, while amplicon studies still dominate the field, how can shotgun metagenomics, multi-omics integration, and reproducible machine learning frameworks transform descriptive community results into mechanistic models and early-warning indicators that can be tested across trials, scenarios, and production stages. In this framework, each omics layer answers a different part of the causal chain: amplicon or metagenomic profiling identifies which microbes and genes are present, metatranscriptomics indicates which microbial or host pathways are active, metabolomics captures biochemical outputs at the gut interface, host transcriptomics records epithelial and immune responses, and phenotypic indicators determine whether these molecular signals correspond to altered gut health, disease resistance, feed response, or resistome risk.

The novelty of this review lies in integrating evidence that is often considered separately, including feed-driven microbiome modulation, mucosal immune regulation, aquaculture stress, antibiotic exposure, resistome ecology, metagenomics, multi-omics, and predictive analytical tools, into a unified framework for salmonid gut-health management. Rather than reviewing microbial taxa, feed additives, immune markers, or ARGs as isolated topics, this review links them as connected components of a production-relevant host–microbe–environment system. This integrated perspective helps distinguish where current evidence is mechanistic, where it remains mainly associative, and how externally validated multi-omics and microbiome-informed indicators could support feed evaluation, health monitoring, disease-risk assessment, and antimicrobial stewardship in rainbow trout and Atlantic salmon.

**Figure 1:**
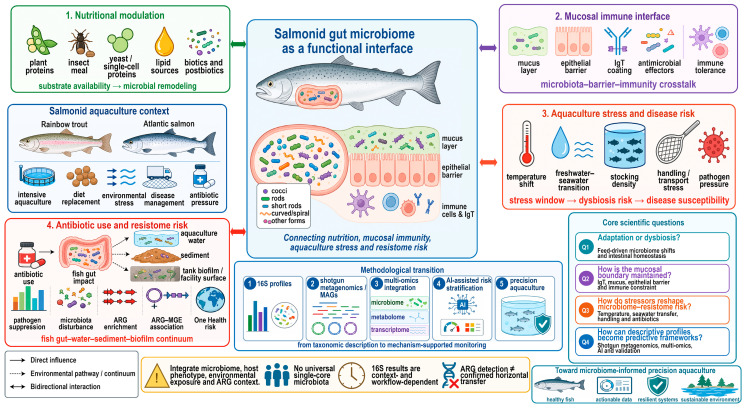
Review framework and core scientific questions for salmonid gut microbiome research. The framework conceptualizes the salmonid gut microbiome as an interface connecting nutritional modulation, mucosal immunity, aquaculture stress and disease risk, antibiotic exposure and resistome risk, methodological transition, and microbiome-informed precision aquaculture. It highlights that microbiome shifts should be interpreted together with host phenotypes, environmental exposures, ARG context, and evidence boundaries rather than as isolated taxonomic patterns. Arrows indicate conceptual links among nutrition, mucosal immunity, aquaculture stress, antibiotic exposure, resistome risk, and precision-aquaculture applications, but they do not imply that every relationship has been causally validated. This figure should be interpreted as an evidence-organizing framework rather than as a confirmed mechanistic pathway.

Therefore, within the conceptual framework proposed in this review (Figure 1), the central aim is not simply to list microbial taxa detected in the intestines of rainbow trout and Atlantic salmon. In Figure 1, the arrow from nutritional modulation to the gut microbiome indicates that feed ingredients and additives can reshape microbial composition and functional potential, whereas the arrows linking the microbiome to mucosal immunity, disease risk, and resistome risk indicate proposed interpretive connections that require different levels of evidence. The figure should therefore be read from left to right as a framework for organizing evidence rather than as a validated causal pathway. Rather, Figure 1 summarizes the major thematic links discussed in this review and should be interpreted as an evidence-organizing framework rather than as a fully validated causal mechanism. It should be emphasized that, at present, there is still a lack of validation across sites and production stages for whether specific taxa, functional genes, or ARGs can stably predict salmonid production performance, the degree of mucosal inflammation, or field-level disease risk. Future research should integrate metagenomics, host phenotypes, environmental exposures, and reproducible machine learning methods to progressively build interpretable and transferable frameworks for monitoring salmonid gut health. The arrows indicate conceptual relationships supported to varying degrees by available evidence and do not necessarily imply direct causal validation for every link.

### 1.4. Literature Search and Selection Strategy

This review was designed as a narrative and critical review rather than a formal systematic review or meta-analysis. Relevant literature was identified through searches of Web of Science Core Collection, PubMed, Scopus, and Google Scholar using combinations of terms including “rainbow trout”, “*Oncorhynchus mykiss*”, “Atlantic salmon”, “*Salmo salar*”, “salmonid”, “gut microbiome”, “intestinal microbiota”, “mucosal immunity”, “nutrition”, “feed substitution”, “probiotic”, “prebiotic”, “antibiotic resistance gene”, “resistome”, “metagenomics”, “multi-omics”, and “precision aquaculture”. To address the broader context of existing aquaculture microbiome reviews, we also considered review-level terms such as “fish and shellfish microbiome”, “host-microbiome interaction”, “fish mucosal immunity”, “aquaculture disease microbiome”, “bioactive compounds”, “environmental factors”, and “functional feed additives”. Broad fish and shellfish reviews were used to define the general background of the field, whereas primary salmonid studies were prioritized when drawing species-specific conclusions for rainbow trout and Atlantic salmon. Priority was given to studies directly involving rainbow trout or Atlantic salmon, followed by studies on other fish or aquaculture systems when they provided relevant mechanistic, methodological, or environmental context. Articles were selected when they addressed gut microbial composition, nutritional modulation, mucosal immune interactions, aquaculture stress, antibiotic exposure, ARGs, MGEs, metagenomic methods, or microbiome-informed aquaculture applications. Evidence from non-salmonid fish, shellfish, environmental microbiomes, wastewater, livestock, or general methodological studies was used only to support mechanistic interpretation or methodological discussion and was not treated as direct salmonid evidence.

### 1.5. Evidence Grading and Interpretation

Because the studies reviewed here differ substantially in species, production stage, sample type, sequencing method, phenotype measurement, and experimental design, we used a qualitative evidence-grading approach to guide interpretation. This grading was not intended as a formal GRADE-style systematic assessment, but as a transparent framework for distinguishing levels of inference. We considered evidence strong when findings were supported by direct salmonid data, biologically relevant phenotypes, appropriate methodological resolution, and consistency across independent studies or production contexts. Evidence was considered moderate when it came from direct salmonid studies but was limited by sample size, species or stage coverage, lack of replication, or incomplete functional validation. Evidence was considered limited when it relied on few studies, indirect evidence from non-salmonid systems, technical prediction, or findings that were strongly dependent on sample type or analytical workflow. We used associative evidence for studies showing correlations or co-variation between microbial features and host, diet, environmental, immune, or disease phenotypes without experimental validation. We reserved causal evidence for studies involving direct manipulation, controlled challenge, gnotobiotic or defined-community models, strain- or metabolite-level validation, or other designs capable of testing whether a microbial feature contributes mechanistically to a host or production outcome. Throughout the review, functional predictions from 16S rRNA data are therefore treated as hypothesis-generating, shotgun metagenomic or metatranscriptomic findings as stronger functional evidence, and experimentally validated host–microbe effects as causal evidence.

### 1.6. Use of AI-Assisted Tools

AI-assisted tools were used only as auxiliary tools during manuscript preparation. For the literature-related work, AI-assisted tools were used to support preliminary literature discovery, assist in generating search terms, and help identify potentially relevant publications. All references identified with the assistance of AI tools were manually checked against the original publications. The final inclusion, exclusion, interpretation, and synthesis of the literature were completed by the authors.

AI-assisted tools were also used to assist in the preparation and modification of some illustrative graphical elements in the figures, including intestine- and fish-related visual components. These tools were used to improve visual clarity, readability, and aesthetic consistency. The conceptual design of the figures, scientific labels, organization of graphical modules, arrangement of arrows, and interpretation of relationships were designed, revised, and verified by the authors. AI-assisted tools were not used to generate primary data, analyze experimental data, fabricate references, create scientific evidence, or produce unsupported conclusions.

## 2. Gut Microbial Community Composition and Its Determinants

Before comparing microbiota composition across rainbow trout and Atlantic salmon studies, it is necessary to distinguish biological variation from sampling and workflow variation. Salmonid gut microbiome studies differ in intestinal segment, digesta versus mucosa sampling, fasting duration, time after feeding, feed controls, DNA extraction, amplified 16S region, sequencing strategy, taxonomic database, and statistical workflow. These differences can change both the apparent dominant taxa and the inferred effects of diet, stress, disease, or antibiotic exposure. Therefore, throughout this section, comparisons among studies are interpreted cautiously, with particular attention to whether the analyzed material represents luminal digesta, mucosa-associated communities, whole-intestine contents, feces, or environmental inputs.

### 2.1. Baseline Composition and Differences Among Studies

Current evidence indicates that the shared feature of the gut microbial communities of rainbow trout and Atlantic salmon is not the dominance of a single genus across all experiments. Rather, major bacterial phyla repeatedly reported across studies include Proteobacteria/Pseudomonadota, Firmicutes/Bacillota, Bacteroidota, and Actinobacteriota. Because many salmonid microbiome studies still use legacy phylum names, this review retains the names reported in the original studies while noting their updated taxonomic equivalents where appropriate [15,16,17,18,19,20,21,22,23]. In rainbow trout, the autochthonous core genera summarized by systematic reviews mainly include *Mycoplasma*, *Aeromonas*, *Clostridium*, *Deefgea*, *Streptococcus*, *Cetobacterium*, *Lactobacillus*, *Lactococcus*, *Methylobacterium*, *Corynebacterium*, *Shewanella*, and *Staphylococcus*. However, these genera are not equally dominant in every study, intestinal segment, or developmental period [15,23]. The early gut microbiota of rainbow trout is highly malleable: first feeding and diet type can markedly alter colonization sequence and subsequent community structure, making direct comparisons among larval-stage studies with different first-feeding schemes difficult [24,25]. In larger or grow-out rainbow trout, studies have reported relatively high abundances of Proteobacteria, Firmicutes, Actinobacteriota, Fusobacteriota, and *Mycoplasma*-associated groups. Thus, “common dominant taxa” in rainbow trout are better understood as a set of lineages that shifts with host status, feed background, stress level, and sampling layer, rather than as a fixed phylogenetic profile [26,27,28,29,30,31,32,33,34,35].

For Atlantic salmon, baseline compositional patterns are somewhat easier to summarize: Firmicutes and Proteobacteria commonly co-dominate in freshwater parr and early seawater stages, whereas the relative abundance of *Mycoplasma* and *Lactobacillus* often increases during middle-to-late seawater phases, and *Photobacterium* may decline [17,18,19,20,21,22,36,37]. High-resolution segmental studies further show that differences among intestinal regions and microenvironments within the same Atlantic salmon can be sufficient to change the ranking of “dominant bacteria”. For example, in a high-resolution study comparing intestinal microenvironments in Atlantic salmon, Proteobacteria accounted for approximately 90% of mucosa-associated communities, whereas digesta samples contained approximately 47% Proteobacteria and 38% Firmicutes [16]. A continuous tracking study of distal–intestinal digesta in Atlantic salmon showed that Firmicutes and Proteobacteria together accounted for approximately 80% of total abundance overall. As fish progressed from post-seawater transfer toward the adult stage, *Lactobacillus* and *Mycoplasma* increased gradually, whereas *Photobacterium* declined relatively [20]. Therefore, when different studies report Proteobacteria, Firmicutes, *Mycoplasma*, *Photobacterium*, or lactic acid bacteria as dominant groups, these findings are often not truly contradictory. Instead, they usually reflect misalignment in fish age, freshwater/seawater stage, intestinal segment sampled, mucosa versus digesta sampling, time after feeding, and taxonomic database version [16,20,22,28,38,39,40,41].

Taken together, the comparative implication is that rainbow trout and Atlantic salmon share several recurrent phylum-level patterns, especially repeated detection of Proteobacteria/Pseudomonadota, Firmicutes/Bacillota, Bacteroidota, and Actinobacteriota, but they differ in the production windows and biological drivers most often linked to microbial variation. In rainbow trout, current evidence more clearly emphasizes early feeding, diet responsiveness, freshwater rearing, handling stress, temperature stress, and family or strain effects. In Atlantic salmon, compositional interpretation is more strongly shaped by freshwater–seawater transition, smoltification, seawater grow-out stage, intestinal microenvironment, and commercial-scale temporal variation. Therefore, dominant taxa reported for the two species should be interpreted as context-dependent microbial signatures rather than as fixed species-specific markers.

### 2.2. Key Factors Shaping Community Structure

Developmental stage is the primary variable explaining differences in gut microbiota between the two salmonids, because rainbow trout already shows rapid colonization and replacement from first feeding to the juvenile stage, while Atlantic salmon likewise exhibits stage-specific microbial signatures from the embryonic period to later growth stages [21,24,25]. Intestinal location and sample type are also highly explanatory. In particular, differences between mucosa and digesta often exceed the effects of some individual dietary treatments, because digesta more readily reflects transient microbes and recent feed inputs, whereas the mucosa more closely represents stably colonized communities [16,28,29,39]. In Atlantic salmon, the transition from freshwater to seawater markedly reshapes community structure, but this remodeling is not a complete “reset”, because stably co-occurring core OTUs can still be observed across freshwater and seawater stages. This suggests that host filtering and environmental exposure act simultaneously as dual forces [17,18,19,20,21].

Feed composition is one of the best-supported and most operationally tractable variables. In rainbow trout, plant proteins, animal by-products, carbohydrate/protein-ratio adjustment, and substitution with different protein sources can all induce community rearrangement; in Atlantic salmon, plant-derived ingredients, functional additives, processed black soldier fly products, and prebiotics can likewise alter community composition and diversity [11,20,25,27,34,35,36,42,43]. However, studies do not agree on whether increased diversity necessarily indicates a better health state. Some treatments mainly alter transient taxa in digesta, whereas others more closely affect mucosal colonizers; moreover, microbial DNA carried by the feed itself can further amplify apparent feed effects in digesta-based results [11,16,20,38,39,42].

Direct evidence for temperature effects is stronger in rainbow trout, because both acute heat stress and sustained high-temperature exposure can simultaneously alter gut microbial structure, metabolic profiles, barrier function, and inflammation-related indices [30,31,32,33]. In contrast, Atlantic salmon studies more often provide field evidence in which season, seawater transfer, feeding intensity, and sampling time are intertwined. For example, time after feeding can significantly alter hindgut fecal scores and gut microbial diversity in summer. Without controlling the sampling time point, seasonal fluctuations can easily be misinterpreted as diet or site effects [20,39].

The effects of stocking density and operational stress on the rainbow trout microbiota do not always exceed host filtering and diet effects. A classic study reported that its large core intestinal microbiota showed some robustness to variation in diet and rearing density; nevertheless, long-term handling stress can interact with dietary background and alter community composition without necessarily changing α-diversity synchronously [26,27]. For Atlantic salmon, direct evidence for an independent main effect of stocking density remains limited, because most commercial-scale studies simultaneously involve seawater transfer, season, feeding, and feed changes. Stocking density has therefore not yet been cleanly separated from co-variables [20,39].

**Figure 2:**
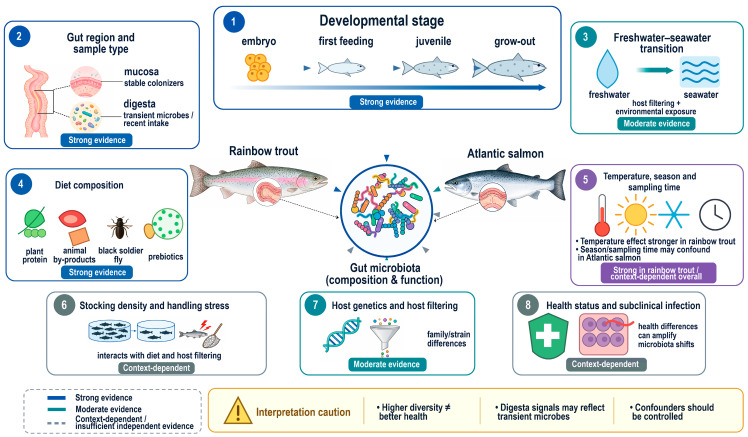
Drivers of gut microbiota composition in salmonids. Developmental stage, gut region and sample type, freshwater–seawater transition, diet composition, temperature, season and sampling time, stocking density and handling stress, host genetics, health status, and subclinical infection jointly shape microbial composition and potential function. The figure emphasizes that evidence strength varies among drivers and that confounders such as feeding history, sample matrix, and health status must be controlled when comparing studies. The drivers shown here may interact within the same production system, and their arrows should not be interpreted as independent one-way effects. Apparent differences in microbiota composition may also reflect sample matrix, intestinal segment, feeding history, health status, and analytical workflow rather than a single biological driver.

Regarding genetic background, family and strain studies in rainbow trout indicate that host genotype can influence the active microbiota or community structure under stress, whereas Atlantic salmon still lacks sufficient family-manipulation experiments. Nevertheless, co-diversification between intestinal *Mycoplasma* and salmonid hosts suggests that host filtering is not a weak process [27,37,44]. In addition, if samples are obtained from fish groups with inconsistent health status or subclinical infection, community differences may be substantially amplified by health problems. These major determinants (Figure 2) indicate that, when defining “baseline composition”, histology, health scoring, and dietary records should be examined alongside microbial profiles, rather than relying solely on 16S relative abundances for ecological interpretation [20,36,45]. In Figure 2, the arrows should be interpreted as interacting drivers rather than independent one-way effects. Developmental stage, gut region, mucosa versus digesta sampling, diet, temperature, stress exposure, host genetics, and health status can overlap within the same fish cohort, which explains why apparent “diet effects” or “disease-associated microbiota” may partly reflect sampling matrix, production stage, or environmental co-variation.

### 2.3. Methodological Comparison and Boundaries of Evidence

From the perspective of study design, 16S rRNA amplicon sequencing remains the main method in gut microbiome research on rainbow trout and Atlantic salmon because it is relatively inexpensive, mature, and suitable for large-sample and multi-treatment designs. It is particularly appropriate for asking whether community structure has changed and which high-abundance taxa are changing [16,17,18,19,20,21,24,25,27,36,40,41]. However, 16S results are strongly affected by primer choice, amplified region, database version, denoising or OTU/ASV workflow, and statistical framework; therefore, relative abundances and taxonomic levels are not inherently comparable across studies [22,38,40,41]. For fish digesta samples, these biases are compounded by microbial DNA carried by feed and by time after feeding. If the research goal is to characterize relatively stable colonizing communities, sampling only the contents without simultaneously sampling the mucosa and feed controls will usually include more transient input signals within what is counted as the “gut microbiota” [16,38,39].

The advantage of shotgun metagenomics is that it moves the question from “who is present” to “what genes and functions these microbes may carry”. It can provide higher species- or genome-level resolution and can directly analyze metabolic pathways, nutrient-transformation potential, virulence factors, and gene-level features such as antibiotic resistance genes [22,29,40,41]. Whole-shotgun studies in rainbow trout have shown functional partitioning between the midgut and hindgut in amino acid synthesis/degradation, protein fermentation, butyrate metabolism, and methanogenesis-related functions, while the Salmon Microbial Genome Atlas for Atlantic salmon further advances functional interpretation to the levels of high-quality genomes, metabolic modeling, and metatranscriptomics [22,29]. Thus, for judgments involving metabolic pathways, functional interactions, virulence factors, or ARGs, functional prediction based solely on 16S can at most serve as a hypothesis-generating approach and cannot replace direct metagenomic or transcriptomic evidence [22,40,41].

At the same time, shotgun approaches involve higher costs and workflow barriers. They require higher DNA quality, greater sequencing depth, host DNA depletion, and more demanding bioinformatic pipelines, and sample size is often limited as a result. This is an important reason why functional-omics studies of these two salmonids remain fewer than 16S-based compositional studies [22,29,40,41]. Based on current evidence, the more prudent evidence hierarchy in this section is as follows: baseline composition, community differences, and environmental responses should primarily rely on large-sample 16S studies, whereas functional analysis, nutrient metabolism, virulence factors, and ARGs should preferably be supported by shotgun metagenomics or metatranscriptomics. If the two types of evidence are inconsistent, differences in sample type and workflow should be examined before advancing strong host-mechanistic interpretations [22,38,39,40,41].

## 3. Effects of Feed Substitution and Nutritional Modulation on the Gut Microbiome

### 3.1. Resource Constraints and the Application Context of Alternative Ingredients

In formulated feeds for Atlantic salmon and rainbow trout, resource constraints on fishmeal and fish oil have shifted formulation optimization from whether to replace them to which ingredients, inclusion levels, and processing methods should be used. Current major routes include plant proteins, insect proteins, yeast or other single-cell proteins, and vegetable or algal oils [46]. Existing salmonid evidence suggests that fishmeal replacement does not necessarily lead to intestinal dysbiosis. For example, when animal by-products partially replace fishmeal in rainbow trout, microbial richness can remain relatively stable; Atlantic salmon also tolerates pea protein concentrate and poultry by-products better than soybean-meal-type formulations with high antinutritional-factor loads [47]. The soybean meal model in Atlantic salmon remains an important reference for this section because the effects of plant replacement are not merely a “change in protein source”. Rather, they arise from the combined effects of antinutritional factors, undigested substrates, mucus-layer stress, and local inflammation; distal enteritis and community rearrangement often occur in parallel [47,48,49]. Therefore, evaluation of alternative ingredients should not rely only on final body weight or feed conversion ratio. It should also integrate microbial structure, intestinal morphology, barrier-related genes, and metabolic signals, because these indicators may emerge before growth differences become evident [50]. In Atlantic salmon, intestinal location, developmental phase, and freshwater–seawater transition can themselves strongly shape the microbiota. Thus, lipid replacement at first feeding may not immediately produce significant differences, whereas ingredient effects are more likely to accumulate when lipid replacement is combined with long-term plant-protein use [46].

In rainbow trout, when fishmeal is fully replaced by plant protein or fish oil is replaced by rapeseed oil, bacterial and fungal α-diversity can still decline even if final body weight does not differ significantly from that of commercial controls. Therefore, “unimpaired weight gain” should not be equated directly with “unimpaired intestinal homeostasis” [51]. Insect proteins and some yeast proteins have attracted attention not because they are necessarily superior to fishmeal, but because their fermentable substrates, cell-wall polysaccharides, and chitin-associated structures differ from those of soybean meal and often induce different trajectories of microbial change [11,43,52,53,54,55,56].

### 3.2. Feed Ingredients Reshape Gut Microbes Through Substrate and Lipid Signals

The primary route by which feed ingredients affect the salmonid gut microbiome is through changing the substrates available in the hindgut. Incompletely digested proteins, polysaccharides, chitin, and their degradation products can reshape competitive relationships among microbial functional groups [50]. In Atlantic salmon, a challenge diet based on 30% soybean meal can shift digesta microbiota toward lactic-acid-bacterial communities; different yeast-processing methods can further modify this “soybean-meal-type” trajectory. For example, inactive yeast generally maintains this pattern, whereas autolysed *Cyberlindnera jadinii* can enrich *Pediococcus* and is accompanied by higher predicted mucus O-glycan degradation pathways [57]. This finding suggests that some single-cell protein preparations, particularly yeast-derived ingredients processed in different ways, should not be interpreted only as nitrogen sources. Instead, their effects may depend on species identity, processing method, cell-wall composition, and the background diet, all of which can influence how intestinal microbes interact with dietary and mucus-associated substrates. Therefore, “yeast protein” or “single-cell protein” should not be treated as a single functionally equivalent category [58]. Insect protein also has relatively clear microbiota-selective effects. After Atlantic salmon consume black soldier fly-related diets, both digesta and mucosal communities can be remodeled, although not necessarily in the same direction. Common outcomes include treatment-associated enrichment of Lactobacillaceae, Actinomyces, or chitinolytic Bacillaceae, while distal–intestinal histology and transcriptomes do not show obvious adverse responses [11,43,56]. These taxa should therefore be interpreted as diet-associated microbial signatures rather than as inherently beneficial markers unless they are accompanied by consistent functional, histological, immunological, or challenge-test evidence.

In rainbow trout, black soldier fly ingredients can increase microbial diversity and raise the abundance of Firmicutes and Actinobacteria while decreasing Proteobacteria as well as *Listeria* and *Campylobacter*; these changes have been interpreted as a community shift toward short-chain-fatty-acid-associated functional taxa [53]. Plant proteins and rapeseed oil often show another type of signal. In triploid rainbow trout, after complete replacement of fishmeal with plant protein or complete replacement of fish oil with rapeseed oil, bacterial and fungal α-diversity decrease simultaneously, and genera such as *Schlesneria*, *Brevundimonas*, *Mycoplasma*, *Verticillium*, and *Aspergillus* become more dominant, whereas some low-abundance taxa appear as compensatory or network-associated elements rather than as confirmed health-promoting taxa [51]. This instability is also evident in the mucus layer of rainbow trout: under handling-stress conditions, plant-protein diets further reduce mucus-layer microbial richness, suggesting that diet effects may interact with host stress status [59]. The interpretation of lipid source requires stratification. In Atlantic salmon at first feeding, complete replacement of fish oil with vegetable oil did not significantly alter gut microbiota composition, indicating that developmental stage may be stronger than the effect of a single lipid source [46].

**Figure 3:**
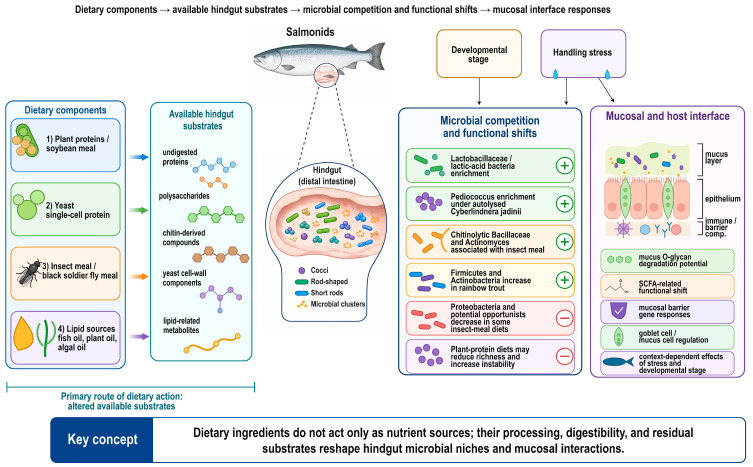
Dietary modulation of salmonid hindgut microbial niches through substrate availability and mucosal-interface responses. Plant proteins, yeast and other single-cell proteins, insect meal, and alternative lipid sources alter the supply of undigested proteins, polysaccharides, chitin-derived compounds, yeast cell-wall components, and lipid-related metabolites. These inputs can reshape microbial competition, functional capacity, mucus and epithelial responses, and host outcomes in a context-dependent manner. The arrows summarize plausible substrate–microbe–mucosal-interface pathways, but taxonomic enrichment or diversity change alone should not be interpreted as beneficial or harmful. Dietary effects require phenotype-aware interpretation together with histology, mucus and epithelial-barrier indicators, immune markers, metabolites, growth, or disease-resistance outcomes.

This does not mean, however, that lipid replacement has no effect on the mucosal interface. In Atlantic salmon, replacing 50% of fish oil with *Schizochytrium* algal oil can increase mucus-cell density in the intestine, skin, and gills, upregulate barrier-related genes such as muc2, muc5ac, and defensin/cathelicidin, and reduce the HSP70 response; 100% replacement did not show the same advantage [60]. As summarized in Figure 3, dietary modulation is interpreted here through a substrate–microbe–mucosal-interface pathway rather than through taxonomic change alone. The key arrows indicate that plant proteins, yeast cell-wall components, insect-derived chitin, and lipid sources may first alter luminal substrates and microbial competition; these microbial changes may then affect metabolite production, mucus-layer use, epithelial responses, and inflammatory tone. However, the thinner or cautionary links in this pathway, especially those involving bile-acid profiles, amino-acid metabolites, and mucus O-glycan utilization, remain less directly validated in salmonids. Direct studies remain fewer than studies of community structure and histology, and the evidence is not yet sufficient to support quantitative mechanistic generalization [50].

Mechanistically, these ingredient-driven changes may act through several substrate–microbe–host pathways. Fermentable carbohydrates, yeast cell-wall polysaccharides, and chitin-derived compounds can provide substrates for microbial fermentation and may increase the production of short-chain fatty acids such as acetate, propionate, and butyrate [35,36,43,58,61]. These metabolites are relevant because they can support epithelial energy metabolism, tight-junction regulation, mucus production, and anti-inflammatory signaling, although direct salmonid evidence remains less developed than in mammalian systems [62,63,64]. Lipid replacement may also affect the gut interface through bile-acid transformation, because bile acids shape microbial selection and can act as host signaling molecules involved in lipid absorption, epithelial stress responses, and inflammatory regulation [30,46,51,60]. In parallel, predicted enrichment of mucus O-glycan degradation pathways after some yeast-derived ingredients suggests a possible interaction between dietary substrates, mucin utilization, and mucus-layer turnover; depending on context, this may reflect adaptive use of host-derived glycans or increased pressure on the mucus barrier [58,59,65]. Chitin-containing insect meals may favor chitinolytic or fermentative taxa, potentially altering SCFA-related metabolism and competitive exclusion of opportunistic bacteria [11,43,53,54]. Finally, microbial amino-acid metabolism, including protein fermentation and pathways related to arginine, tryptophan, and branched-chain amino acids, may influence epithelial repair, oxidative stress, and immune tone [30,62,63,66]. Therefore, the most informative interpretation of feed-induced microbiome remodeling is not simply whether specific taxa increase or decrease, but whether substrate availability, microbial metabolic output, mucus and epithelial barrier responses, inflammatory markers, and host growth or disease-resistance phenotypes change in a coherent direction [7,20,49,67,68].

### 3.3. Health-Management Value of Biotics and Postbiotics

Compared with ingredient replacement, probiotics, prebiotics, and postbiotics are more directly aligned with health-management goals, including promoting beneficial bacterial colonization, limiting the expansion of opportunistic pathogens, and mitigating mucosal-barrier injury. They are therefore better understood as non-antibiotic microbial-management tools rather than as simple nutritional supplements [36,68,69,70,71,72,73,74]. In Atlantic salmon, oligosaccharide prebiotics can alter microbial communities in both the distal intestine and the skin, suggesting that microbial modulation may occur across multiple mucosal surfaces rather than being limited to the intestinal lumen [70]. Long-term experiments in adult Atlantic salmon also indicate that prebiotics do not always markedly improve histological and inflammatory indicators. More stable changes are often reflected in temporal community trajectories, such as reduced α-diversity, increased *Mycoplasma*, and decreased LAB including *Lactobacillus*, *Leuconostoc*, and *Lactococcus* [36]. Thus, prebiotic effects depend on the application window, host stage, and basal diet and should not be evaluated as a simple binary variable of “added or not added” [70]. For probiotics, supplementation with fish-derived LAB can make *Lactobacillus* the dominant genus in the distal intestine of Atlantic salmon and alter network structures in both digesta and mucus-layer communities, indicating that the effect extends beyond composition to community interactions [72].

In a soybean-meal-induced enteritis model, combined supplementation with *Lactobacillus plantarum* and *L. fermentum* increased lactic acid, propionic acid, succinic acid, and acetoacetic acid in intestinal chyme, reduced lamina propria width, and increased supranuclear vacuoles. Its effect should therefore be interpreted as combined regulation of metabolic and barrier phenotypes rather than as simple “bacterial addition” [71]. In rainbow trout, continuous or cyclic feeding of autochthonous *L. plantarum* R2 can increase intestinal lactic-acid-bacterial colonization. Cyclic feeding upregulates CD8, TGF-β, IL8, and TLR9 without inducing additional increases in IL1 and TNF-α, suggesting that feeding rhythm influences regulatory effects [73]. Evidence on postbiotics is currently concentrated mainly in rainbow trout. Postbiotics derived from lactic-acid-bacterial fermentation can increase microbial diversity and improve resistance to *Lactococcus garvieae*, while *Weissella cibaria*-derived postbiotics can increase lactic-acid-bacterial counts and improve survival after *Yersinia ruckeri* challenge [68]. Accordingly, the most prudent positioning of salmonid biotic products is as auxiliary modules for stabilizing the microbiota and buffering enteritis risk in low-fishmeal and low-fish-oil diets, rather than as interventions that can reliably reproduce growth-promoting effects in all formulation contexts. Direct evidence for synbiotics and Atlantic salmon postbiotics remains insufficient, and firm conclusions are not yet warranted [69]. To clarify the strength and limitations of these nutritional interventions, Table S1 summarizes the main intervention categories, salmonid species, production context, sample type, microbiota response, host phenotype, and evidence limitation. This summary highlights that the strongest interpretation comes from studies in which microbiota changes are evaluated together with histological, barrier, immune, metabolic, growth, or challenge-test endpoints, whereas microbiota-only studies remain more context-dependent.

Overall, Table S1 shows that nutritional strategies differ substantially in evidence strength. Interventions supported only by taxonomic shifts, diversity changes, or predicted functional profiles should be interpreted as hypothesis-generating. Stronger support is available when microbiota remodeling is accompanied by coherent host endpoints, such as intestinal histology, mucus-cell dynamics, epithelial-barrier markers, immune-gene expression, metabolite profiles, growth performance, feed-utilization traits, or pathogen-challenge outcomes. Therefore, plant proteins, insect meals, yeast products, lipid replacements, prebiotics, probiotics, synbiotics, and postbiotics should not be ranked simply by whether they increase microbial diversity or enrich selected taxa. Their relevance depends on whether microbial changes align with measurable host benefit, absence of mucosal burden, or improved disease resistance under a defined production context.

## 4. Interactions Between the Gut Microbiome and Mucosal Immunity

### 4.1. The Mucus Layer, Epithelial Barrier, and Secretory Immunity

The salmonid intestine is responsible for both nutrient digestion/absorption and mucosal immune defense. Its mucosa-associated lymphoid tissue is mainly diffuse, and the epithelium, mucus cells, B/T cells, and innate immune effector molecules jointly form a local immune interface that continuously handles dietary antigens and commensal bacterial loads [75,76]. The mucus layer is not an inert coating but a mucin-dominated dynamic barrier. It can physically reduce bacterial attachment and also participate in host–microbe recognition through glycan structures; therefore, its integrity directly affects the spatial separation between the microbiota and the epithelium [65].

In rainbow trout, intestinal bacteria are coated predominantly by IgT rather than IgM, indicating that IgT is the dominant secretory immunoglobulin in intestinal mucosal homeostasis, whereas IgM more likely represents a mucosal complement to systemic humoral immunity [4,77]. Also in rainbow trout, the secretory component formed by cleavage of the polymeric immunoglobulin receptor can coat most intestinal bacteria in vivo, suggesting that the “mucus-pIgR/SC-immunoglobulin” axis is a continuous barrier module that cooperatively restricts bacterial adhesion to the epithelium [78]. A more mechanistic interpretation is that IgT-mediated coating may act as a selective immune-exclusion system at the salmonid mucosal surface [3,4,77]. Polymeric IgT transported across epithelial cells through pIgR, together with the secretory component, can be retained in the mucus layer and bind subsets of commensal bacteria, opportunistic taxa, or pathogens [4,77,78]. For commensals, this coating may help maintain mucosal tolerance by permitting microbial persistence while limiting direct epithelial contact and excessive inflammatory activation [5,77,79]. For opportunistic bacteria or pathogens, stronger or altered IgT coating may promote spatial segregation within the mucus, reduce adhesion to epithelial surfaces, and limit translocation across the barrier [4,77,78]. Thus, IgT does not only mark bacterial exposure; it may help organize the physical and immunological boundary between tolerated microbiota and potentially harmful microbes [76,77,79]. Together with mucus glycans, epithelial tight-barrier function, antimicrobial peptides, and innate inflammatory signaling, IgT-mediated immune exclusion provides a plausible mechanism by which the salmonid intestine maintains homeostasis and improves resistance to infection [65,66,80,81]. Antimicrobial effects in the salmonid intestine do not depend solely on immunoglobulins. Antimicrobial peptides such as cathelicidin, hepcidin, β-defensin, and histone-derived peptides also help inhibit colonization by potential pathogens. Thus, intestinal microbial homeostasis is essentially maintained by the combined action of microbes, the mucus/epithelial barrier, secretory immunoglobulins, and antimicrobial molecules [76,80].

### 4.2. Microbial Regulation, Immune Maturation, and Disease Resistance

Current salmonid evidence indicates that gut microbes and mucosal immune maturation progress in parallel rather than changing independently. During development from late freshwater to seawater stages in Atlantic salmon, expression of intestinal immune and barrier-related genes generally increases, while *Mycoplasma* in adult samples is positively associated with barrier genes and *Leuconostoc* is positively associated with sets of immune genes [36,67]. In rainbow trout feeding-intervention studies, *Pediococcus acidilactici* MA18/5M can simultaneously reshape the gut microbiota and stimulate intestinal immunity, and multi-omics studies further show that probiotic-driven changes in the microbiota and metabolites can be accompanied by improved growth, immune phenotypes, and higher resistance to *Aeromonas salmonicida* [63,82].

At the cellular level, the rainbow trout RTgutGC epithelial model shows significant increases in il1b, il6, il8, and tnfa after LPS stimulation, and functional ingredients such as MOS can modulate its barrier and inflammatory responses. This suggests that the intestinal epithelium itself is an active immune unit that senses microbe-associated molecules and integrates PRR-related signals [66]. Mechanistically, microbial-associated molecular patterns, including LPS, peptidoglycan, flagellin, unmethylated CpG DNA, and fungal or yeast-derived polysaccharides, may be detected by salmonid intestinal epithelial cells and resident immune cells through TLRs and other PRRs [66,75,76,79]. Activation of these receptors can recruit adaptor pathways such as MyD88 and subsequently stimulate NF-κB and MAPK signaling, leading to the transcription of pro-inflammatory mediators such as il1b, il6, il8, and tnfa [5,66,67,81]. In parallel, cytosolic PRR and inflammasome-related pathways may contribute to caspase-dependent maturation of IL-1β/IL-18-like inflammatory responses, whereas viral or nucleic-acid-associated signals can activate interferon-regulatory pathways and induce interferon-stimulated antiviral genes [75,83,84,85]. These epithelial and immune-cell responses are also linked to the production of antimicrobial effectors, including cathelicidin, hepcidin, β-defensin, lysozyme, and histone-derived peptides, which help limit pathogen colonization and maintain the spatial separation between luminal microbes and the mucosal surface [75,76,79,80]. In an IHNV infection model, crude shiitake mushroom polysaccharide intervention simultaneously improved rainbow trout gut microbial structure, SCFA-related metabolism, and immune-barrier status, and reduced IHNV-related mortality. This provides relatively direct experimental support in salmonids for the logical chain of “microbiota modulation-barrier stabilization-inflammation reduction-enhanced disease resistance” [85].

**Figure 4:**
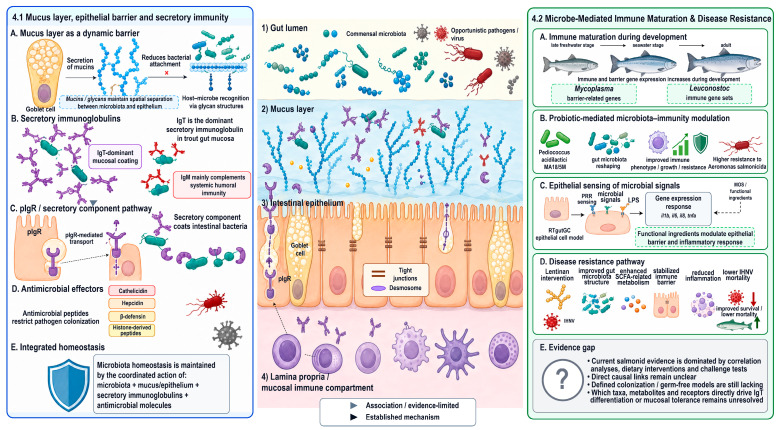
Microbiota–mucosal immune interactions in salmonids. The salmonid gut interface integrates luminal microbes, the mucus layer, intestinal epithelium, secretory immunoglobulins, antimicrobial effectors, and lamina propria immune cells. Current evidence supports coordinated roles of mucus-barrier function, IgT-associated bacterial coating, pIgR/secretory-component transport, antimicrobial peptides, epithelial sensing, immune maturation, probiotic-mediated modulation, and disease-resistance pathways, while direct strain–metabolite–immune causal links remain insufficiently validated. The schematic highlights the mucosal interface as an integrated system involving mucus, epithelium, IgT, pIgR/secretory component, antimicrobial molecules, immune signaling, and microbial communities. Arrows represent coordinated host–microbe interactions supported to different degrees by current evidence; direct strain–metabolite–immune causal pathways remain insufficiently validated in salmonids.

If we further ask which colonizing bacteria, which metabolites, and which receptors directly drive IgT differentiation, mucosal tolerance programs, and the broader mucus-barrier and disease-resistance modules outlined here (Figure 4), current salmonid research still relies mainly on correlation analysis, nutritional intervention, and challenge experiments, and lacks germ-free or defined-community colonization models. Therefore, this causal chain should still be regarded as insufficiently supported [36,63,76].

### 4.3. Dysbiosis, Enteritis, and Pathogen Resistance

When pathogen infection, enteritis, or environmental stress disrupts the above homeostasis, salmonid intestinal dysbiosis usually manifests as simplified community structure or excessive expansion of dominant taxa, impaired mucosal barrier function, upregulated inflammatory factors, and reduced pathogen resistance. However, not all models show a fully consistent decrease in α-diversity; a more cautious formulation is therefore “community restructuring with increased opportunistic-pathogen risk” [27,31,83,84,86,87,88,89,90]. In this review, dysbiosis is therefore not interpreted as a single-direction causal label. In salmonid enteritis models, it is more appropriately viewed as a context-dependent state that may arise as a consequence of epithelial and mucus-barrier disturbance, while also acting as an amplifier of inflammation and pathogen susceptibility once the altered microbial community becomes established. In rainbow trout, for example, IHNV immersion infection can invade the digestive tract and intestinal mucosa, induce increased mucus production and local antiviral and antibacterial responses, and significantly alter the gut microbial community; differences in infection status on farms likewise lead to clear stratification of rainbow trout gut microbiota [83,84,86]. Acute heat stress and long-term handling stress provide environmental evidence for a continuum of “stress-barrier injury-microbiota remodeling-inflammatory amplification”, because they can simultaneously damage rainbow trout intestinal epithelial structure and permeability, alter pro-inflammatory factors and metabolites, and reconstruct dominant gut taxa [27,31].

In Atlantic salmon, soybean meal- or saponin-associated enteritis can increase the attached bacterial load and community differences, induce epithelial stress, apoptosis, and mucosal inflammation, and clearly distinguish bacterial community structure between healthy and inflamed intestines. Importantly, removing only three groups of soybean-protein antinutritional factors does not automatically eliminate soybean-induced enteritis, indicating that the “feed factor-microbiota-mucosal immunity” relationship is not a linear effect of a single toxic molecule [87,88,89,90]. Conversely, lactic acid bacteria or functional formulations can, to some extent, narrow the lamina propria, increase mucus cells or innate defense indicators, and shift the microbiota toward healthy controls. However, histological inflammation is not completely reversed in some trials; therefore, “microbiota modulation” for salmonid enteritis is better understood as a barrier-immune regulatory strategy rather than a single repair method that can reliably replace control of the underlying cause [71,81,91,92].

## 5. Aquaculture Stress, Antibiotic Use, and Risks of Resistance-Gene Transmission

### 5.1. Aquaculture Stress, Disease Management, and Demand for Medication

In salmonids such as Atlantic salmon and rainbow trout, the intestine is not merely a digestive organ. It is a mucosal immune interface jointly composed of the mucus layer, epithelial barrier, local immune cells, and commensal microbes; therefore, any persistent aquaculture stress can simultaneously alter barrier integrity and microbial homeostasis [75,93]. High density, crowding, temperature fluctuation, capture/grading, seawater transfer, and hypoxia are not independent management variables. Through increased cortisol, osmotic reconstruction, altered energy allocation, and changes in mucosal secretion, they jointly shape a “disease-susceptibility window” in salmonids [18,93,94,95,96,97,98].

Among these stressors, seawater transfer is a relatively well-supported critical window. During the freshwater-to-seawater transition in Atlantic salmon, dominant gut taxa can shift from freshwater-type core communities toward seawater-stage dominant groups such as *Lactobacillus* and *Photobacterium*; multiple intestinal and systemic immune transcriptional indicators are also downregulated, suggesting that “seawater transfer-microbiota rearrangement-immune vulnerability” may occur in parallel [17,18,95,96]. Hypoxia and higher water temperature may amplify this vulnerable state. In Atlantic salmon, chronic hypoxia can induce neutrophil infiltration and changes in inflammatory-factor profiles in the intestinal mucosa, while in rainbow trout, heat stress can further rearrange the gut microbiota and its metabolic network, translating environmental stress into intestinal inflammation and metabolic imbalance [31,32,97,98].

For transport, grading, and repeated capture, original studies that directly pair commercial salmonid management scenarios with gut microbiota outcomes remain insufficient. However, in rainbow trout experimental models, daily handling stress alone is sufficient to alter both intestinal-content and intestinal-mucus microbiota, indicating that routine management operations themselves may trigger dysbiosis [27,59]. Once disease outbreaks occur, accumulated stress can reinforce infection-associated dysbiosis. *Tenacibaculum* infection is associated with changes in the distal–intestinal microbiota of Atlantic salmon, and different susceptibility of rainbow trout to *Flavobacterium psychrophilum* is likewise accompanied by distinguishable gut microbial differences. Thus, aquaculture stress is not background noise in medication use, but an important upstream variable determining disease-management intensity and demand for antimicrobials [45,99,100,101].

### 5.2. Dual Effects of Antibiotics on the Gut Microbiome

In salmonid aquaculture, antibiotics still serve the practical function of suppressing bacterial disease and reducing acute mortality risk in the short term, especially in high-loss settings such as bacterial cold-water disease and salmonid rickettsial disease, where florfenicol and tetracyclines remain common tools in treatment chains [101,102,103]. From an intestinal-ecological perspective, however, this short-term benefit is often accompanied by longer-term costs. In Atlantic salmon, OTC exposure can significantly reduce gut microbial diversity and allow tetE-carrying *Aeromonas* to become a dominant resistant population, directly showing that “pathogen control” and “ecological niche release” can coexist during the same treatment period [104]. Subsequent studies further show that this disturbance is not limited to expansion of a single resistant bacterium. After antibiotic feeding in Atlantic salmon, dominant intestinal bacterial phyla shift markedly, and in rainbow trout the gut community remains unstable 14 days after drug withdrawal. These findings suggest that salmonid gut responses to antibiotics are not one-off shocks but community-succession processes that may extend into the withdrawal period [105,106]. Mechanistically, antibiotic exposure can reshape the intestinal ecological niche through selective suppression and niche release. Susceptible commensals may be depleted during treatment, reducing colonization resistance and freeing adhesion sites, nutrients, and mucus-associated substrates that can be used by resistant or antibiotic-tolerant taxa. In parallel, antibiotic-induced disturbance of microbial competition may alter the availability of fermentation products, bile-acid-related metabolites, and host-derived substrates, thereby changing the conditions under which ARG-carrying populations persist. Thus, enrichment of resistant taxa should be interpreted not only as direct selection by the drug, but also as an ecological consequence of altered community structure and delayed microbiota recovery.

The effects of florfenicol in Atlantic salmon are not limited to community structure. After feeding, gastrointestinal transcriptional responses involve pathways such as apoptosis, DNA metabolism, LXR/RXR and FXR/RXR activation, and protein ubiquitination, suggesting that antibiotic treatment acts simultaneously on host tissues and intestinal microbial ecology [107]. Selection pressure during treatment may also spill over to the system level. In fish models, OTC can increase tetA in the distal intestine and tank biofilm; florfenicol exposure can simultaneously elevate ARGs and MGEs at the fish-gut metagenomic level and increase the likelihood that ARGs enter mobile-element contexts [108,109]. This is important because ARGs located near or within plasmids, integrons, transposons, insertion sequences, or integrase/transposase-associated regions may be more likely to persist under repeated selection than ARGs occurring only as isolated chromosomal markers. Plasmids can maintain ARGs across bacterial lineages when they are stably replicated or partitioned, integrons can capture and express resistance cassettes, and transposons or insertion sequences can rearrange resistance regions within and between replicons. Therefore, antibiotic exposure may increase resistome risk not only by enriching ARG abundance, but also by favoring genetic contexts in which ARGs are maintained with mobile or recombinational elements. Therefore, antibiotic effects on the salmonid gut should be understood as a “dual effect”: antibiotics can reduce pathogen pressure in the short term, but they may also compress commensal diversity, delay community recovery, and increase ARG mobilization potential, thereby raising the probability of resistant-bacterial expansion and recurrent dysbiosis.

### 5.3. ARG Transmission Networks and One Health Risk

From a resistome perspective, salmonid aquaculture systems should not be viewed only as an “inside-the-fish-gut” issue. They should instead be understood as connected transmission networks linking the fish intestine, water, sediment, uneaten feed/fishmeal, and biofilms on facility surfaces. The connectivity among these compartments can arise through several non-mutually exclusive routes. Feces, sloughed mucus, and released intestinal bacteria can carry ARGs from the gut into water and sediment; uneaten medicated feed and fishmeal-derived organic matter can introduce nutrients, residual antibiotics, and microbial DNA into the environment; biofilms on tanks, nets, pipes, and other facility surfaces can concentrate bacteria, extracellular DNA, and MGEs; and sediments can act as longer-term sinks where organic loading and low diffusion may prolong resistance-associated selection. These interfaces may therefore create ecological “meeting points” where resistant bacteria, susceptible recipients, extracellular ARGs, and mobile genetic elements coexist. At the fish end, long-term or repeated use of florfenicol and OTC on salmon farms can select for multidrug-resistant intestinal bacteria and is accompanied by frequent ARG detection, indicating that the fish gut itself can form a persistent resistance reservoir [110].

At the environmental end, fishmeal and uneaten feed are not passive background materials. Sediment experiments show that fishmeal itself can promote ARG amplification, and in “fishmeal + tetracycline” systems, the main effect driving resistome growth may even be more strongly associated with nutrient input than with the antibiotic itself. This indicates that organic loading can amplify the ecological basis of resistance selection [111,112]. Farm effluent can transport resistance pressure into open waters. Studies of fish-farm discharge from Jeju Island and other aquaculture-impacted waters suggest that ARG-rich resistomes can occur in aquaculture effluents or related waters. Thus, the farm-to-water dissemination chain has an observational basis in the field, although comparisons across regions, media, and farming modes still require stratification [113,114].

**Figure 5:**
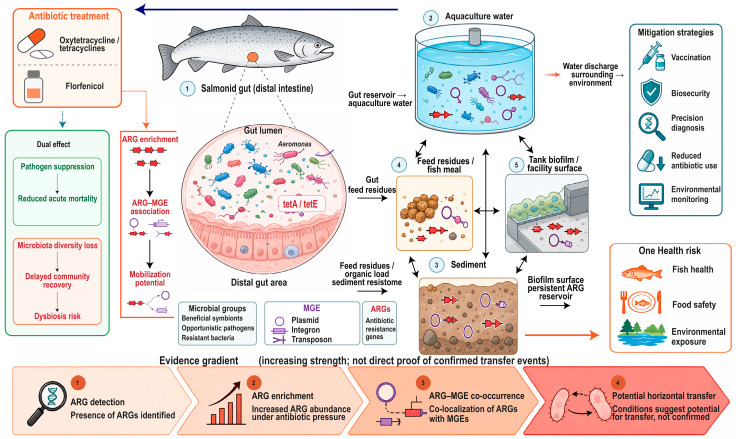
Antibiotic exposure, ARG enrichment, ARG-MGE association, and One Health risk in salmonid aquaculture. Antibiotics may suppress pathogens and reduce acute mortality, but they can also disturb commensal communities, delay recovery, enrich ARGs, and increase the mobilization potential of resistance determinants. The figure distinguishes ARG detection, ARG enrichment, ARG-MGE co-occurrence, and inferred transfer potential across the fish intestine, water, sediment, feed residues, and biofilm continuum. Arrows indicate possible ecological connectivity and mobility-related risk across the fish intestine, water, sediment, feed residues, and biofilms. ARG detection, ARG enrichment, or ARG–MGE co-occurrence should not be interpreted as confirmed horizontal gene transfer unless host-resolved, plasmid-resolved, or time-resolved transfer evidence is available.

Nevertheless, it is essential to distinguish among several levels of evidence: “ARG detection”, “ARG enrichment”, “co-localization of ARGs with MGEs”, and “inferred horizontal transfer”. Detection of shared ARGs between water and fish intestine indicates only the presence of a cross-media network and potential exchange; it should not be automatically equated with a tracked HGT event. Current higher-level evidence mainly comes from metagenomic results showing concurrent increases or physical proximity of ARGs with MGE-associated elements such as integrases and transposases in antibiotic-exposed fish intestines [109,115]. Within the ecological continuum linking antibiotic exposure, ARG enrichment, ARG-MGE association, and One Health risk (Figure 5), salmonid antibiotic management should not only ask whether treatment is effective. It should also assess whether the fish gut forms a resistance reservoir, whether environmental dissemination routes emerge, and whether resistance determinants enter more mobile genetic contexts. Recent reviews, global literature surveys, and regional meta-analyses all regard aquaculture AMR as a coupled issue involving animal health, food safety, and environmental exposure, and identify vaccination, biosecurity, precision diagnosis, reduced antimicrobial use, and environmental monitoring as priority mitigation pathways [103,116,117,118].

## 6. From Microbiome to Resistome: The Need for Metagenomic Research

### 6.1. Limitations of 16S rRNA Amplicon Sequencing

In salmonids and their aquaculture environments, the main advantages of 16S rRNA amplicon sequencing are its low cost, mature workflow, and suitability for comparing community profiles across many samples. Its resolution and interpretive power are also affected by primer selection, amplified region, and amplification bias; therefore, results are not inherently comparable across studies [119]. In a multi-platform comparison of recirculating aquaculture systems, Rieder et al. showed that short 16S amplicons can distinguish sample type, rearing unit, and temporal change, but primer choice and amplicon length systematically affect diversity estimates, the number of annotatable taxonomic units, and parts of the taxonomic composition. Thus, 16S is more suitable as an ecological screening tool for “who is here” than as an evidence layer for “which risk genes these microbes carry” [119].

More importantly, 16S does not directly measure functional genes. Methods such as PICRUSt and PICRUSt2 infer function based on reference genomes and phylogenetic proximity, and their outputs should be regarded as indirect predictions rather than direct evidence [120,121]. A 2024 limitations assessment further noted that 16S-based metagenome prediction and functional profiling can be biased when reference-database coverage is insufficient, phylogenetic relationships deviate from assumptions, or community complexity increases. These approaches therefore cannot replace direct observation of functional genes, resistance genes, and mobile genetic elements [122].

For salmonid systems, this issue is particularly pronounced. Dominant intestinal commensals are not static genus-level labels; they show host-associated diversification and subgroup-level functional differences. Taking *Mycoplasma* as an example, salmonid-associated lineages show signals of host co-diversification and potential mutualistic metabolic traits. Remaining at the 16S genus-level conclusion can therefore oversimplify the true strain–host relationship [37,123]. Accordingly, reliance on 16S alone cannot adequately evaluate functional changes, ARG types, potential hosts, or transmission risk in salmonid aquaculture systems, especially because it cannot answer the core risk-assessment question of whether an ARG is located on a plasmid, integron, transposon, or genome of a potential pathogen [124,125].

### 6.2. Advantages of Shotgun Metagenomics

The central advantage of shotgun metagenomics is that it analyzes composition, function, resistance, mobility, and host background within the same DNA dataset, thereby providing information on species composition, metabolic pathways, ARGs, MGEs, viruses/phages, and potential hosts at the same time [22,119,126,127]. In fish-intestine cases, Tyagi et al. demonstrated that shotgun approaches not only yield finer taxonomic profiles than 16S but also simultaneously identify metabolic pathways and ARG composition, converting “community change” into a joint interpretation of “functional change” and “resistance change” [126].

In salmonid research, Rasmussen et al. recovered a MAG of the dominant *Mycoplasma* through genome-resolved metagenomics and inferred its potential mutualistic functions; Vera-Ponce de León et al. constructed a high-quality genome catalogue and functional map of the Atlantic salmon gut, indicating that salmonid gut research has entered a stage in which “genome-function-host health” can be interpreted jointly [22,123]. Similarly, multi-omics and whole-shotgun studies in rainbow trout have shown that prebiotic/synbiotic interventions and midgut–hindgut anatomical partitioning induce different metabolic potentials and functional divisions. Such findings depend on real functional genes rather than 16S prediction and are therefore more persuasive for feed evaluation and gut-health interpretation [29,62].

At the aquaculture-environment level, shotgun metagenomics can incorporate fish intestine, water, and sediment into the same resistance-ecology framework. Xu et al. inferred potential hosts of tet(X) and blaCTX-M in a comparison of aquaculture modes in China; Tian et al. observed that 75% of ARG-bearing contigs in non-intensive aquaculture systems also carried MGEs and recovered 475 high-quality MAGs, of which 81 carried ARGs. This information is beyond what 16S can directly provide [127,128]. At the tool level, CARD 2023, ResFinder 4.0, MEGARes 2.0, ARGs-OAP v2.0/v3.0, and DeepARG were designed for different purposes. Salmonid studies should not treat databases as interchangeable “black boxes”; instead, they should combine tools according to specific aims such as acquired ARG detection, resistance-mutation identification, environmental resistome quantification, or deep learning-based recognition [129,130,131,132,133,134].

### 6.3. Host–ARG–MGE Coupling as the Next Key Step

The decisive methodological transition in this field is not merely the replacement of 16S with shotgun metagenomics, but the shift from “microbiome composition” to “host-ARG-MGE coupling”. Transmission risk can be evaluated more meaningfully only when the bacterial host of an ARG, its mobile-element context, and its potential occurrence in pathogen-associated genomes are resolved, rather than inferred from abundance differences alone [125,127]. Existing methods already provide a route: Hi-C can link plasmids, integrons, and ARGs to host chromosomes; long-read sequencing can span repetitive regions and reconstruct continuous structures connecting ARGs with neighboring MGEs; single-cell isotope probing and targeted metagenomics can connect “active resistant phenotypes” with hosts and mobile elements; and methods such as Argo have advanced species-resolved host tracking in long-read samples [135,136,137,138,139].

**Figure 6:**
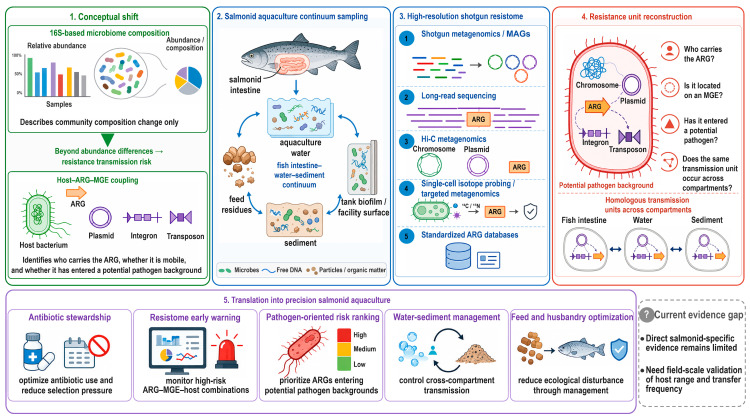
Roadmap from descriptive microbiome profiling to host–ARG–MGE coupling and precision salmonid aquaculture. The workflow progresses from 16S-based community profiling to continuum sampling, shotgun metagenomics and MAGs, long-read sequencing, Hi-C, targeted approaches, standardized ARG databases, and resistance-unit reconstruction. Downstream applications include antibiotic stewardship, resistome early warning, pathogen-oriented risk ranking, water–sediment management, and feed or husbandry optimization, while current salmonid-specific evidence gaps are acknowledged. This roadmap represents a proposed research and monitoring progression rather than a currently established routine workflow. The transition from descriptive microbiome profiling to precision salmonid aquaculture requires standardized sampling, host–ARG–MGE linkage, multi-omics integration, causal validation, and external testing across sites, diets, seasons, and production stages.

For salmonid aquaculture systems, future priorities (Figure 6) should therefore shift from asking whether the microbiota changes after a treatment to asking whether specific ARGs occur in potential pathogens, whether they are co-localized with plasmids, integrons, or transposable elements, and whether homologous resistance units appear across the fish intestine–water–sediment continuum. This is where resistance ecology intersects directly with aquaculture health management [22,124,127,135,136,137]. However, studies that use Hi-C, long-read sequencing, or single-cell technologies to assign ARGs to specific salmonid intestinal hosts or pathogens remain scarce. At present, high-resolution direct evidence comes more often from broader aquaculture environments, wastewater, soil, or other animal samples; therefore, the actual host range and field transfer frequency of ARGs within salmonid systems remain insufficiently resolved [124,127,135,136,137,138,139]. Rather than continuing to infer “potential functions” from 16S, salmonid research should systematically deploy shotgun-centered frameworks supported by MAGs, long reads, Hi-C, and standardized ARG databases, upgrading the research endpoint from “microbiome change” to the source, host, mobility, and risk level of resistance units [124,125,127,129,130,131,132,133,134,135].

### 6.4. Open Questions and Boundaries of Evidence

Current evidence is sufficient to show that 16S alone cannot support functional and resistance-risk assessment in salmonids, whereas salmonid-specific ARG host tracking remains substantially behind community-structure description [22,124,127]. The strongest direct evidence currently comes from functional metagenomics, MAGs, and multi-omics studies in Atlantic salmon and rainbow trout, but closed-loop resolution of continuous transmission units across the fish intestine–aquaculture water–sediment interface remains limited [22,29,62,124,127]. Thus, strengthening host–ARG–MGE coupling is a well-supported methodological priority. However, existing evidence is not sufficient to claim that any particular ARG class has undergone high-frequency field transfer within salmonid systems; such statements should remain cautious [124,127,135,136,137,138,139].

## 7. Applications of Multi-Omics and Artificial Intelligence in Salmonid Gut-Health Research

### 7.1. Analytical Value of Multi-Omics Integration

The core value of multi-omics integration is not the simple stacking of sequencing platforms, but the placement of feed composition, gut microbial structure, microbial function, metabolite flux, and host mucosal responses into a single framework to determine which microbes affect which host phenotypes through which functional pathways [62]. A practical multi-omics framework should therefore be organized by biological questions rather than by platform names. 16S rRNA profiling asks whether community structure has changed; shotgun metagenomics asks which microbial genes, metabolic pathways, ARGs, MGEs, and potential hosts are present; metatranscriptomics asks which microbial or host pathways are transcriptionally active under a given diet, stressor, infection, or antibiotic exposure; metabolomics asks which biochemical products accumulate at the gut interface; host transcriptomics asks how epithelial, immune, inflammatory, stress, and barrier pathways respond; and phenotypic indicators determine whether these molecular changes correspond to altered histology, mucus-barrier status, feed utilization, growth, mortality, or disease resistance. Salmonid research has gradually moved from simple 16S descriptions toward coordinated analyses of metagenomics, metabolomics, and host transcriptomics. Representative evidence comes from probiotic/synbiotic trials and heat-stress models in rainbow trout, as well as high-resolution functional-omics studies in Atlantic salmon [22,31,32,62,124,140]. For example, combined 16S, metagenomic, and untargeted-metabolomic analyses of rainbow trout intestinal contents showed that feed additives not only altered community composition, but were also accompanied by changes in arginine biosynthesis, terpenoid-backbone biosynthesis, and lipid-, bile-acid-, and steroid-related metabolite profiles. This indicates that feed effects can now be extended from “taxonomic change” to “functional and metabolic output” [62].

In infection-recovery scenarios, integrated analysis after rainbow trout probiotic intervention and *Yersinia ruckeri* challenge suggests that changes in host immune-gene expression and microbiota remodeling are not independent. Correlative modeling can propose candidate biomarkers related to infection status and recovery, but these still require validation in external cohorts [141]. Heat-stress studies provide another line of evidence. Acute heat stress can be accompanied by intestinal morphological damage, increased barrier permeability, oxidative imbalance, and elevated pro-inflammatory factors; coordinated changes in microbes and metabolites suggest that intestinal microecology may participate in abnormal metabolite transport across the barrier [31]. A more comprehensive three-omics study in rainbow trout advanced this chain to the level of host transcriptional regulation, showing parallel expansion of Enterobacteriaceae, disruption of lipid and amino-acid metabolism, activation of PPAR-α-related signaling, and intestinal injury under heat stress. Thus, the chain of “environmental stress-microbial dysbiosis-metabolic disturbance-host transcriptional response” already has a traceable omics evidence base [32].

In Atlantic salmon, genomic and functional-resource studies show that the core salmon gut microbiota has the potential to degrade feed-derived fibers and release vitamins and other exometabolites, providing a microbial functional map for explaining differences in feed response [22]. A subsequent Communications Biology study used 16S, metatranscriptomics, targeted metabolomics, and host-organ transcriptomics in a salmon mannan-feeding trial, but found limited effects under conventional conditions. This indicates that the value of multi-omics lies not only in identifying effective interventions, but also in excluding formulation strategies that lack functional matching [124]. In addition, a hologenomic study of 460 Atlantic salmon linked gut metagenomes, metabolomes, host transcriptomes, and host genotypes, suggesting that parasite-infection risk is related to host–microbiota interactions rather than being determined by the abundance of a single genus [140].

Therefore, for salmonid gut-health research, multi-omics integration can already connect feed, microbes, metabolites, and host immunity. However, “full-chain” studies that simultaneously cover metagenomes, metatranscriptomes, metabolomes, host transcriptomes, and immune phenotypes, and that have been externally validated across multiple sites, remain relatively scarce [32].

### 7.2. Roles of Machine Learning and Deep Learning

The most realistic role of machine learning and deep learning in this field is not to replace experimental validation, but to screen stable features from high-dimensional, collinear, and batch-effect-prone multi-omics data and convert these features into testable hypotheses for health stratification [142]. For this reason, models intended for gut-health prediction should not rely only on relative abundances of selected taxa. More informative models should integrate taxonomic features with pathway-level metagenomic profiles, metatranscriptomic activity, metabolite modules, host immune or barrier indicators, environmental metadata, and phenotypic outcomes, followed by external validation across farms, diets, seasons, and production stages. SIAMCAT-related research points out common problems in microbiome machine learning, including overly optimistic model evaluation, insufficient cross-study generalization, and inadequate control of confounders. These problems are further amplified in salmonid aquaculture by diet, freshwater–seawater transition, site differences, and sampling batches [142]. Stable feature-selection research also shows that the importance ranking obtained from a single model run is not equivalent to a stable biomarker. Workflows such as RFE need to be used together with stability assessment to improve the reproducibility of microbial feature selection [143].

Returning to salmonids specifically, rainbow trout infection-recovery studies have attempted to jointly model microbiota data and host immune genes and have proposed candidate markers for monitoring infection and recovery. However, this work still mainly represents single-cohort exploration and remains some distance from deployable production models [141]. In broader aquaculture practice, current machine learning applications are still concentrated on fish identification, biomass estimation, behavior analysis, and water-environment prediction, whereas models using gut multi-omics as the core input remain relatively uncommon [144]. A 2025 early-warning system for *Cryptocaryon irritans* disease in mariculture showed that, when multiple years of outbreak records and continuous environmental data are available, RF and XGBoost models can form week-scale, spatially explicit risk-forecasting platforms. This provides a technical reference for incorporating salmonid gut indicators into future disease-prediction models, but it does not mean that gut multi-omics early warning is already mature in application [145].

For ARGs, DeepARG and HMD-ARG improve ARG recognition and annotation in metagenomic sequences from the perspectives of deep neural networks and hierarchical multi-task learning, respectively; the latter can also provide information on resistance class, resistance mechanism, and mobility-related features [134,146]. ProteinBERT-like models have advanced ARG mechanism recognition to the level of protein language models and can retain some discriminatory ability for low-homology sequences. This has methodological value for the many ORFs in salmonid gut metagenomes that remain poorly annotated, but the results still need to be interpreted together with experimental and ecological evidence [147]. At a broader level of functional interpretation, deep learning protein-annotation frameworks and DeepFRI can improve inference efficiency from sequence or structure to function, potentially alleviating the problem of excessive unknown functional fragments in salmonid gut metagenomes [148,149]. Meanwhile, methods such as mmvec, mixOmics/DIABLO, and MOFA provide a tool basis for joint learning of “microbe-metabolite” or “multi-omics-phenotype” relationships. Compared with simple correlation networks, they are better suited for feature compression, identification of covarying modules, and prioritization of mechanisms [150,151,152,153].

Caution is still required. Based on the currently verifiable public literature, “deep-learning-based health-state classification”, “individualized prediction of feed response”, and “ARG enrichment risk stratification” in salmonid gut contexts remain mainly at the stage of methodological transfer potential or single-scenario exploration, and have not yet formed mature external-validation systems. Therefore, they should not be described as mature applications [124,134,146,147].

### 7.3. Application Boundaries for Early Warning and Risk Monitoring

A realistic future application is to incorporate enteritis early warning, disease-risk prediction, feed-response evaluation, and ARG enrichment-risk monitoring into a single stratified decision-making framework, although the maturity of these tasks differs [52,124,134,146,147]. For enteritis early warning, the existing salmonid literature shows that disease state, external infection, parasite pressure, and adverse environments can be projected onto gut microbiota, metabolites, and mucosal responses. A more feasible strategy is therefore not to rely on the absolute abundance of a single genus, but to construct a composite deviation score based on “individual historical baseline + key functional pathways + immune/barrier indicators” [141]. For disease-risk prediction, environment-driven models in mariculture have demonstrated the feasibility of week-scale forecasting. At the current stage, salmonid research is better positioned to overlay gut microbial and host indicators onto environmental time series to form a three-layer “environment-host-microecology” risk model, rather than judging risk based solely on water temperature or salinity [145].

For feed-response evaluation, insect meal, plant-based diets, prebiotics, and high-carbohydrate formulations can all induce observable microbial and metabolic remodeling, but host endpoints reported across studies are not fully consistent. Therefore, future modeling should shift from asking whether a formulation is effective on average to asking which fish will benefit and which fish will experience intestinal burden [35,52,61,62,154,155]. For ARG monitoring, a more implementable workflow is to combine shotgun-derived ORFs, ARG prediction results, mobile-element context, core microbiota structure, and host inflammation indicators into site-specific risk panels. Deep learning can be used to improve recognition of ARGs and unknown proteins, but it cannot independently replace ecological and phenotypic validation [134,146,147,148,149].

Elements that may currently be regarded as having an application basis include candidate microbiota/pathway screening, assisted classification of infection status, environment-driven disease forecasting, and ARG/protein functional annotation. In commercial settings, salmonid enteritis early warning, individualized feed recommendation, and ARG enrichment-risk dashboards are still more appropriately classified as future potential applications [134,141,146,147]. Therefore, the field should not pursue “fully automated precision aquaculture” prematurely. A more prudent path is to use multi-omics and AI first as a decision-support layer for prioritizing samples, compressing key indicators, and warning of high-risk batches. Before external validation across sites, seasons, and diets is completed, describing these approaches as mature production tools remains insufficiently supported [142,144,145].

## 8. Outlook: From Experience-Based Aquaculture to Microbiome-Driven Precision Aquaculture

### 8.1. Longitudinal Cohorts and Standardized Sampling

Publicly comparable salmonid 16S data integrations currently cover 19 studies and 783 samples, but differences in amplified regions, DNA-extraction procedures, sample types, and metadata records substantially limit direct cross-study comparability [7]. In the few genuinely longitudinal studies, the same Atlantic salmon cohort shows clear community remodeling from freshwater RAS production to one week and four weeks after seawater transfer; another commercial-scale study observed continuing intestinal community changes from late freshwater to one year after sea entry. Thus, time itself is a major confounder in salmonid gut microbiome interpretation [12,22].

Microbial DNA carried by feed can also influence digesta-based microbiota profiles. Without paired feed, mucosal, and environmental controls, an apparent “feed-induced microbiota change” may partly reflect feed-carried microbial DNA rather than true host colonization [39]. Hindgut mucosal swabs in rainbow trout can provide mucosa-associated community information through a non-lethal approach, offering a practical route for repeated follow-up of the same fish. However, this method has not yet become a standardized workflow in salmonid research.

More broadly, salmonid studies remain dominated by associations between microbiota and phenotypes, whereas direct evidence is still insufficient for assigning specific strains, pathways, and host backgrounds to defined outcomes. Future work should therefore establish longitudinal microbiome databases stratified by developmental stage, freshwater–seawater transition, feed scheme, aquaculture environment, disease status, and medication history, while recording feed intake, weight gain, feed conversion ratio, mortality, histopathology, and mucosal immune indicators within the same data structure [12,22]. Sampling SOPs should define intestinal segment, digesta versus mucosa, fasting duration, and paired feed, inflow/outflow water, biofilm, and sediment samples. Negative and positive controls and a minimum metadata template are also needed to keep technical batch effects within a controllable range [7,39].

### 8.2. Salmonid-Specific Reference Resources, Multi-Omics, and Causal Validation

Salmonid-specific reference development should build on the Atlantic salmon gut Salmon Microbial Genome Atlas and subsequent bacterial–viral genome catalogues, while integrating cultured isolates, MAGs, phages, functional genes, ARGs, and metabolic annotations into a common framework. This would help transform “16S ASVs” into verifiable functional units [23]. Multi-omics integration should move beyond single-shotgun surveys toward time-series designs linking metagenomes, metatranscriptomes, metabolomes, and host immune phenotypes, because cross-layer temporal information is more likely to reveal functional signals that precede pathological deterioration.

At the analytical level, the value of artificial intelligence and mechanistic modeling is not to generate more complex black-box classifiers, but to convert interpretable links among “feed-microbiota-metabolites-host phenotype” into prediction rules with transfer potential. Existing frameworks in community-scale metabolic modeling, precision nutrition, and AI provide methodological routes for this strategy. Causal validation should follow a stratified pathway from cohort screening of stable associated taxa to SalmoSim or other in vitro functional validation, controlled infection or nutritional challenge, and multisite pilot testing. This is a feasible route for moving from microbiome association toward mechanism and application. For functional-taxa screening, research should prioritize recurrent core members with genomic functional evidence and culture or consortium potential, such as salmonid-associated *Mycoplasma* lineages and their functional loci. However, development of such taxa into translatable precision probiotics still requires stronger causal evidence [23,38,124].

### 8.3. Precision Nutrition, Health Early Warning, and Resistance-Risk Monitoring

The endpoint of gut microbiome research in rainbow trout and Atlantic salmon should extend beyond weight gain or feed conversion ratio to support antibiotic reduction, health early warning, and environmental risk control. This is where precision aquaculture differs most clearly from experience-based management [46]. The shift is consistent with One Health, because long-term AMR trends in Asian aquatic animals, aquaculture-environment risk frameworks, and resistome reviews all indicate that antimicrobial use, ARG enrichment, MGE dissemination, and effluent discharge form a continuum linking farmed animals, the environment, and human exposure [117,118]. Future salmonid precision-aquaculture platforms should therefore incorporate ARG monitoring into routine workflows and should track resistance genes, host bacteria, and mobile elements across fish intestine, feed, water, sediment, and effluent, followed by joint analysis with drug exposure, disease events, and production indicators.

At present, published evidence indicates that salmonid microbiome-based early-warning and precision-nutrition models are still moving from proof of concept toward external validation. Transferable tools across sites, seasons, and feed systems remain scarce, and direct evidence from rainbow trout and Atlantic salmon is still insufficient for routine deployment [46]. Only when longitudinal databases, standardized sampling, salmonid-specific microbial genome catalogues, multi-omics integration, causal validation, functional-taxa screening, and ARG risk monitoring are developed together can salmonid aquaculture move from experience-driven management toward microbiome-driven precision aquaculture [23,117].

## 9. Discussion

### 9.1. Reframing the Salmonid Gut Microbiome as a Dynamic Functional Interface

The present synthesis should be interpreted against the background of existing reviews on fish and shellfish microbiomes. Those reviews have established that diet, environment, mucosal tissues, disease challenge, and antimicrobial exposure can shape aquatic animal microbiomes. However, broad taxonomic syntheses necessarily combine species with different gut anatomies, life histories, thermal niches, feeding ecologies, culture systems, and immune characteristics. For salmonids, species-level synthesis is particularly necessary because freshwater–seawater transition, smoltification, cold-water culture, fishmeal and fish-oil replacement, temperature sensitivity, and antibiotic-reduction pressures interact in ways that cannot be fully resolved by generalized fish-and-shellfish frameworks.

A central implication of the literature reviewed here is that the gut microbiome of rainbow trout and Atlantic salmon should not be interpreted as a static taxonomic inventory, but as a dynamic functional interface linking diet, mucosal physiology, environmental exposure, and disease susceptibility. Recurrent phyla and genera can certainly be identified across studies, and some lineages appear repeatedly enough to justify discussion as common or recurrent members of salmonid intestinal communities. However, the available evidence does not support the existence of a single universally stable core microbiota that is conserved across developmental stages, freshwater and seawater production phases, intestinal regions, sample matrices, and farming systems [7,15,16,17,18,19,20,21,22,23]. Instead, “core” should be understood more cautiously as a context-dependent set of recurrent taxa shaped by host filtering within specific ecological and production windows.

This distinction is not semantic. It directly affects how results are interpreted. Early colonization after first feeding, freshwater-to-seawater transfer, intestinal regionalization, and the contrast between digesta and mucosa all generate meaningful biological variation in community structure [16,17,18,19,20,21,24,25]. In addition, feed composition, temperature, stress exposure, and health status further modulate the microbiome, while technical factors such as DNA extraction, amplified 16S region, and reference database choice can alter the apparent taxonomic outcome [7,22,38,39,40,41]. Accordingly, reports describing *Mycoplasma*, lactic acid bacteria, *Photobacterium*, Proteobacteria, or Firmicutes as “dominant” are often not contradictory findings but snapshots of different host states, sampling layers, and analytical workflows.

These considerations also argue for restraint when using terms such as “dysbiosis” or “health-associated taxa.” In salmonids, dysbiosis should not be reduced to deviation from a single idealized community structure, nor should health be inferred from the abundance of any one genus in isolation. A taxon that appears beneficial in one production stage or sample type may mark adaptation, transient substrate availability, or even compensatory instability in another. Thus, the most informative framing is not whether the salmonid gut microbiome matches a universal reference profile, but whether microbiome remodeling is accompanied by coherent changes in histology, barrier integrity, mucosal immunity, metabolism, disease resistance, and production outcome [7,16,20,22,28,39,40,41].

### 9.2. Nutritional Modulation and the Limits of Taxon-Based Interpretation

Nutritional intervention is one of the most tractable routes for manipulating the salmonid gut microbiome, but it is also one of the most vulnerable to overinterpretation. Plant proteins, insect meals, yeast and other single-cell proteins, lipid replacement strategies, and functional additives such as prebiotics, probiotics, and postbiotics can all reshape community composition in rainbow trout and Atlantic salmon [10,11,34,35,36,42,43,46,47]. Yet microbial change per se should not be equated with health improvement. Increases or decreases in diversity, enrichment of lactic acid bacteria, or altered abundances of *Mycoplasma* and other recurrent taxa may reflect adaptive reorganization, altered substrate flow, mucosal stress, or feed-derived DNA signals, depending on context [11,16,38,39,50].

This is particularly important in the context of fishmeal and fish-oil replacement. A persistent weakness in interpretation is the tendency to treat unaffected growth performance as evidence that intestinal homeostasis has also been preserved. The reviewed studies do not justify that conclusion. In salmonids, unchanged body weight or feed conversion may coexist with altered microbial diversity, changes in mucus-associated communities, modified barrier-related gene expression, or histological signals that suggest subclinical intestinal burden [46,50,51]. Conversely, some alternative ingredients, including insect-derived products and selected yeast preparations, may induce substantial microbiome remodeling without detectable adverse histological effects, indicating that microbial restructuring can also reflect functional adaptation rather than pathology [11,43,56,57,58]. The key point is that microbial shifts require phenotypic interpretation; they do not provide a sufficient endpoint on their own.

The same caution applies to biotics and postbiotics. Prebiotics, probiotics, and postbiotics may help buffer the intestinal ecosystem, limit expansion of opportunistic taxa, or modulate metabolic outputs and immune phenotypes, particularly under low-fishmeal or inflammatory dietary contexts [36,68,69,70,71,72,73,74]. However, their effects are not universal and are strongly conditioned by host developmental stage, basal diet, dosing schedule, and the ecological baseline of the intestinal community. Therefore, the most robust interpretation of nutritional modulation is integrative: taxonomic data should be read together with distal–intestinal histology, mucus-cell dynamics, epithelial-barrier markers, inflammatory tone, microbial or host metabolites, disease-challenge performance, and production traits. Without that framework, taxon-based interpretation risks overstating both benefit and harm.

### 9.3. Mucosal Immunity, Dysbiosis, and Disease Susceptibility

Mucosal immunity is the most plausible mechanistic bridge connecting microbiome remodeling with host phenotype in salmonids. The mucus layer, epithelial barrier, secretory immunoglobulin system, antimicrobial peptides, and inflammatory signaling pathways together define the ecological rules of intestinal colonization [4,65,75,76,77,78,80]. In rainbow trout, the preferential coating of intestinal bacteria by IgT and the involvement of pIgR/secretory component suggest that mucosal immune exclusion and microbial containment are not secondary phenomena, but core organizing forces of gut homeostasis [4,77,78]. In this framework, the microbiome is not simply “present” at the mucosal surface; it is selected, constrained, and tolerated through an active immune-barrier interface.

Current salmonid evidence broadly supports a close relationship between microbiota remodeling and immune phenotype. Across development and nutritional intervention, microbial shifts are associated with variation in barrier-related genes, cytokine profiles, mucus characteristics, and disease resistance [36,63,66,67,82,85]. Likewise, enteritis models and pathogen challenges indicate that community restructuring, barrier perturbation, and inflammation often occur together [71,81,83,84,85,86,87,88,89,90,91,92]. However, most of this evidence remains correlational or inferential. Even when a dietary additive shifts the microbiota, changes specific metabolites, and improves histology or challenge survival, it remains difficult to determine whether the microbiota is driving the immune phenotype, responding in parallel to host physiology, or both. Apart from the gnotobiotic rainbow trout work showing microbiota-mediated protection against *Flavobacterium columnare*, direct causal validation in salmonids is still limited [6].

This distinction becomes especially important under aquaculture stress. Temperature fluctuation, seawater transfer, hypoxia, repeated handling, grading, transport, high-density production, and disease exposure should be viewed as interacting ecological disturbances rather than isolated stressors [17,18,27,31,32,93,94]. Together, they may create a disease-susceptibility window in which mucus secretion, epithelial permeability, immune allocation, and microbiome stability are perturbed simultaneously. In such states, “dysbiosis” is best understood not as a simple loss of diversity, but as a shift toward reduced ecological resilience and increased opportunistic-pathogen risk [27,31,83,84,86,87]. Importantly, alpha-diversity does not decline in every model, and therefore diversity loss alone is not a reliable definition of intestinal dysfunction. What matters more is whether stress-associated microbiome remodeling coincides with impaired barrier function, inflammatory amplification, or reduced capacity to resist infection. Future work will need gnotobiotic systems, defined consortia, and strain-resolved functional validation to move beyond association toward explicit strain–metabolite–immune mechanisms.

### 9.4. Antibiotic Exposure, Resistome Risk, and One Health Implications

Antibiotics occupy a difficult but unavoidable position in salmonid aquaculture. In the short term, they remain important tools for reducing mortality during bacterial outbreaks, particularly when pathogen pressure coincides with stressful production conditions [101,102,103]. Yet from an ecological perspective, antibiotic treatment is also a major disturbance event. Available evidence indicates that antimicrobial exposure may reduce microbial diversity, alter dominant community members, delay post-treatment recovery, and favor enrichment of resistant taxa or resistance determinants within the intestinal ecosystem [104,105,106,107,108,109]. These effects should not be construed as arguments against all therapeutic use, but they do indicate that antibiotic treatment and microbiome stability are often in tension.

The key issue is that antibiotic action is dual. Therapeutically, treatment may relieve acute bacterial pressure; ecologically, it may compress commensal diversity and open selective space for resistant or opportunistic bacteria [104,105,106,107]. Some studies further suggest that antibiotic exposure can increase the frequency with which ARGs and MGE-associated elements are detected together, thereby raising concern about mobilization potential [108,109]. However, this evidence still requires careful wording. Detection of ARGs is not equivalent to confirmed horizontal gene transfer, and even co-localization with plasmid- or transposon-associated signatures does not by itself demonstrate successful transmission across hosts in the field [109,115]. What it does indicate is that the intestinal ecosystem has shifted into a configuration in which resistance maintenance and movement may become more plausible.

From a One Health perspective, the salmonid resistome should therefore not be treated as an internal fish-gut issue alone. Rather, it should be understood within a connected intestine–feed–water–sediment–biofilm–effluent network [13,14,110,111,112,113,114]. Feed inputs, organic loading, farm biofilms, and effluent dispersal may all shape where ARGs accumulate and how long resistance-associated ecological pressure persists. For this reason, future risk assessment should move beyond simple ARG abundance and focus instead on host–ARG–MGE coupling: which bacterial hosts carry the ARGs, whether those ARGs are linked to plasmids, integrons, or transposons, and whether these assemblies occur in potential pathogen backgrounds [124,127,135,136,137,138,139]. At present, direct salmonid-specific evidence at this level remains limited, which is precisely why claims about transmission risk should remain cautious. The priority is not to infer confirmed spread prematurely, but to generate the genomic evidence needed to distinguish reservoir potential from demonstrated transfer.

### 9.5. Methodological Advances from 16S Profiles to Host–ARG–MGE Coupling

Much of the current interpretive tension in the field is methodological. 16S rRNA amplicon sequencing has been indispensable for establishing broad community patterns in Atlantic salmon and rainbow trout, especially in large-sample comparisons across feeds, stages, and environments [7,16,17,18,19,20,21]. It remains highly valuable for answering ecological questions about community turnover and relative differences among cohorts. At the same time, its limitations are especially relevant in salmonids, where primer choice, amplified region, DNA extraction strategy, database version, bioinformatic workflow, and sample type can each alter the apparent community profile [7,22,38,40,41,119]. In digesta-based studies, recent feeding history and feed-derived microbial DNA may further confound interpretation if feed controls and fasting time are not standardized [38,39].

As a result, 16S is well suited to asking “who is present,” but it cannot directly answer which functional genes are carried, which ARGs are present, whether mobile elements are involved, or which host genomes those genes belong to [120,121,122,123,124,125]. Functional inference from 16S-based prediction tools may be useful for hypothesis generation, but it does not substitute for direct evidence. This limitation becomes decisive when the research question concerns virulence, metabolic potential, or resistance risk. Under those circumstances, taxonomic profiles alone are insufficient.

Shotgun metagenomics, MAG reconstruction, long-read sequencing, Hi-C, metatranscriptomics, and metabolomics collectively shift the field from descriptive community profiling toward functional and mechanistic interpretation [22,29,62,123,124,125,126,127,128,129,130,131,132,133,134,135,136,137,138,139]. Genome-resolved resources such as the Salmon Microbial Genome Atlas make it increasingly possible to identify recurrent salmon-associated lineages at genome level and to infer their metabolic roles more specifically than was previously possible with genus-level 16S assignments [22]. The next major transition should therefore not be defined simply as “more shotgun sequencing,” but as a move toward explicit host–ARG–MGE coupling and multi-layered host–microbe interpretation. That transition, however, will only be convincing if coupled with standardized sampling design, adequate sequencing depth, host-DNA management, and transparent analytical workflows. Otherwise, methodological sophistication may outpace biological inference.

### 9.6. Multi-Omics, AI, and the Cautious Path Toward Precision Aquaculture

The long-term promise of salmonid microbiome research lies in integration rather than in any single platform. Multi-omics approaches can begin to connect feed composition, microbial community structure, microbial function, metabolite output, and host mucosal response within a common analytical frame [22,29,31,32,62,124,140]. This is particularly valuable in salmonids, where the same taxonomic shift may correspond to adaptive remodeling in one context and intestinal stress in another. By integrating metabolomics and host transcriptomics with microbiome data, it becomes more feasible to distinguish taxonomic change from functional deviation and to identify signals that precede overt enteritis, poor feed response, or increased disease susceptibility.

Artificial intelligence and machine learning may contribute to this transition, but their realistic role is narrower than is sometimes implied. In the current state of the field, AI is better positioned as a tool for feature screening, risk stratification, hypothesis generation, and decision support than as a mature stand-alone production technology [124,134,140,141,142,143,144,145,146,147,148,149,150,151,152,153]. The major challenge is not the ability to fit a classifier within a single cohort, but the ability to maintain performance across sites, seasons, feeds, developmental stages, and technical batches. This is precisely where microbiome ML often fails if confounding is not handled rigorously and external validation is absent [142,143].

For salmonid gut-health research, the most credible near-term applications are therefore modest but useful: early warning for enteritis risk, comparative evaluation of feed-response patterns, probabilistic disease-risk stratification, and monitoring of conditions associated with ARG enrichment [134,141,145,146,147,148,149]. Even these applications require caution. A model trained in one farming system may capture site-specific signatures rather than transportable biology, and a high-ranking microbial feature is not automatically a stable biomarker. Accordingly, AI should be embedded within a validation pipeline that includes mechanistic interpretation, external cohort testing, and feasibility assessment under commercial conditions. Precision aquaculture, in this sense, is not the automation of microbiome analysis; it is the disciplined use of microbiome-informed evidence to support better nutritional, health, and antimicrobial-management decisions.

### 9.7. Remaining Knowledge Gaps and Future Research Priorities

The most urgent gap in the field is the lack of longitudinal, standardized, ecosystem-aware study design. Future work should prioritize cohort structures that follow fish across developmental stages, smoltification, freshwater-to-seawater transition, dietary changes, and treatment histories, rather than relying primarily on cross-sectional contrasts [7,12,22]. Sampling must be standardized with respect to intestinal segment, digesta versus mucosa, fasting time, and accompanying controls from feed, water, sediment, and biofilm [7,39]. Without such design discipline, it will remain difficult to distinguish biological drivers from technical and temporal noise.

A second major priority is the development of salmonid-specific reference resources. Genome catalogues derived from salmon-associated isolates and MAGs should be expanded and linked to functional annotation, ARG databases, phage information, and host metadata [22,23]. This would improve taxonomic resolution, reduce dependence on generic reference spaces, and make strain-level hypotheses more testable. In parallel, ARG host tracking in salmonid systems should increasingly adopt shotgun metagenomics, MAGs, long-read sequencing, and Hi-C so that risk assessment can move from abundance-based description toward direct host–ARG–MGE assignment [124,127,135,136,137,138,139].

Finally, the field should move from association-based biomarker hunting toward causal validation and translational relevance. This means integrating microbiome, resistome, metabolome, mucosal immune phenotype, farm management variables, and environmental exposure data within reproducible analytical frameworks, then testing hypotheses in controlled nutritional or infection models, defined-community systems, and multisite validations [62,124,140,141,142,143,144,145,146,147,148,149,150,151,152,153]. The broader objective is not merely to catalogue salmonid gut microbes more precisely, but to embed microbiome-informed management within antibiotic reduction, fish welfare, environmental risk control, and a One Health framework. Framed in this way, the salmonid gut microbiome becomes less a descriptive endpoint and more a systems-level interface through which sustainable aquaculture can be interpreted and, eventually, more carefully managed. This perspective provides the necessary context for the concluding synthesis that follows. To make these recommendations more actionable, we propose six priority directions for future salmonid gut microbiome research. First, standardized salmonid gut sampling protocols should be established, including harmonized definitions of intestinal segment, digesta versus mucosa sampling, fasting duration, feeding-time records, paired feed and environmental controls, negative and positive controls, DNA/RNA extraction procedures, sequencing workflow, and minimum metadata reporting. Second, longitudinal freshwater-to-seawater studies should follow the same cohorts across first feeding, smoltification, seawater transfer, seawater grow-out, dietary transitions, disease episodes, and antibiotic treatment history, while recording growth, feed conversion, mortality, histology, mucus-barrier status, and immune indicators. Third, causal validation should be strengthened through defined-community, gnotobiotic, SalmoSim or other in vitro gut models, controlled nutritional challenges, infection models, metabolite supplementation, and strain-level testing, so that microbial associations can be separated from true mechanisms. Fourth, resistome research should prioritize host–ARG–MGE linkage analysis using shotgun metagenomics, MAGs, long-read sequencing, Hi-C, plasmid reconstruction, and standardized ARG databases to determine which bacterial hosts carry ARGs, whether these ARGs occur in mobile genetic contexts, and whether resistance units are shared across the fish intestine, feed, water, sediment, biofilm, and effluent continuum. Fifth, machine learning models should be validated across multiple farms, seasons, diets, production stages, sequencing batches, and geographic contexts, with explicit control of confounders and reporting of external-validation performance rather than only within-cohort accuracy. Sixth, intervention trials should combine microbiome profiling with host health outcomes, including epithelial and mucus-barrier markers, immune-gene expression, metabolomics, histology, growth performance, feed utilization, disease-challenge survival, and ARG enrichment risk, so that nutritional or microbial-management strategies can be judged by integrated biological outcomes rather than by taxonomic shifts alone.

## 10. Conclusions

Overall, the gut microbiome of rainbow trout and Atlantic salmon is best understood as a dynamic functional interface rather than a static taxonomic inventory. It links diet, epithelial and mucus-barrier function, mucosal immunity, aquaculture stress, disease susceptibility, antibiotic exposure, and antimicrobial-resistance risk. Current evidence does not support a single universal core microbiota that remains stable across developmental stages, freshwater–seawater transition, intestinal segments, sample matrices, feed regimes, and production systems. Instead, microbial community structure is shaped by interacting biological, environmental, nutritional, and methodological factors. Gut health or dysbiosis should therefore not be inferred from the increase or decrease in selected bacterial genera alone.

Nutritional modulation is one of the most practical routes for managing the salmonid gut microbiome, but its interpretation must remain phenotype-aware. Plant proteins, insect meals, yeast and other single-cell proteins, lipid replacement, prebiotics, probiotics, and postbiotics can reshape microbial communities; however, microbial shifts alone cannot be equated with improved health or intestinal dysfunction. More reliable evaluation requires joint assessment of microbiota composition, functional pathways, metabolites, mucus and epithelial barrier status, histology, immune markers, disease resistance, and production performance.

Mucosal immunity provides the key biological context for interpreting microbiome remodeling. The mucus layer, epithelial barrier, IgT, pIgR/secretory component, antimicrobial peptides, and inflammatory signaling jointly maintain intestinal homeostasis and regulate the boundary among commensals, opportunistic bacteria, and pathogens. Existing studies suggest close links between microbiota remodeling and immune phenotype, but many findings remain correlational or intervention-based. Causal claims about specific strains, metabolites, or immune programs require strain-resolved analysis, defined-community or gnotobiotic systems, metabolite validation, and controlled challenge experiments.

Antibiotic use extends this field into a One Health framework. Therapeutic antibiotics may reduce bacterial disease pressure in the short term, but they can also disturb commensal communities, delay recovery, and promote ARG enrichment or ARG-MGE association. ARG detection should not be equated with confirmed horizontal transfer. Future risk assessment should move beyond abundance profiles toward host–ARG–MGE coupling across the fish intestine–feed–water–sediment–biofilm–effluent continuum, clarifying bacterial hosts, mobile genetic contexts, and potential pathogen backgrounds.

Future work should prioritize six directions: standardized salmonid gut sampling and metadata protocols; longitudinal cohorts across freshwater-to-seawater production stages; defined-community, gnotobiotic, in vitro, nutritional-challenge, and infection models for causal validation; host–ARG–MGE linkage analysis using shotgun metagenomics, MAGs, long reads, Hi-C, and plasmid reconstruction; externally validated multi-site machine learning models; and intervention trials that link microbiome shifts to histology, mucus-barrier status, immune markers, metabolites, growth, disease resistance, and ARG enrichment risk. These priorities provide a practical route from descriptive microbiome profiling toward mechanism-supported precision nutrition, disease early warning, antibiotic stewardship, and One Health-oriented resistome management.

## Data Availability

No data was used for the research described in the article.

## Supplementary Materials

The following supporting information can be downloaded at: https://www.mdpi.com/article/10.3390/biology15131066/s1, Table S1: Summary of nutritional interventions, microbiota responses, host endpoints, and evidence limitations in salmonid gut microbiome studies.

## Abbreviations

The following abbreviations are used in this manuscript:

AMR	Antimicrobial resistance
ARG	Antibiotic resistance gene
MGE	Mobile genetic element
MAG	Metagenome-assembled genome
HGT	Horizontal gene transfer
Hi-C	High-throughput chromosome conformation capture
RAS	Recirculating aquaculture system
OTC	Oxytetracycline
LAB	Lactic acid bacteria
IgT	Immunoglobulin T
pIgR	Polymeric immunoglobulin receptor
SC	Secretory component
IHNV	Infectious hematopoietic necrosis virus
MOS	Mannan oligosaccharide
SCFA	Short-chain fatty acid
RTgutGC	Rainbow trout gut epithelial cell line
CARD	Comprehensive Antibiotic Resistance Database
ARGs-OAP	Antibiotic Resistance Genes Online Analysis Pipeline
MEGARes	MEGARes antimicrobial-resistance database
DeepARG	Deep learning approach for predicting antibiotic resistance genes
HMD-ARG	Hierarchical multi-task deep learning for annotating antibiotic resistance genes
PICRUSt	Phylogenetic Investigation of Communities by Reconstruction of Unobserved States
PICRUSt2	Phylogenetic Investigation of Communities by Reconstruction of Unobserved States 2
SIAMCAT	Statistical Inference of Associations between Microbial Communities And host phenoTypes
DIABLO	Data Integration Analysis for Biomarker discovery using Latent cOmponents
MOFA	Multi-Omics Factor Analysis
RFE	Recursive feature elimination
RF	Random forest
XGBoost	Extreme Gradient Boosting
