# Supplementary material for: Gut Microbiomes of Rainbow Trout and Atlantic Salmon: Nutritional Modulation, Mucosal Immunity, and Resistome Risk
Source: Biology (Basel). 2026 Jul 3;15(13):1066. doi: 10.3390/biology15131066 (PMC13360211; doi:10.3390/biology15131066)
Supplement: Supplementary file 1 [file biology-15-01066-s001.zip › biology-4376045-supplementary.pdf]

Table S1: Summary of nutritional interventions, microbiota responses, host endpoints, and evidence limitations in salmonid gut microbiome studies.

Intervention type	Species	Life stage / production context	Sample type	Main microbiota change	Host phenotype or functional endpoint	Evidence limitation
Animal by-products or alternative protein sources	Rainbow trout; Atlantic salmon	Juvenile or grow-out stages, depending on study	Intestinal contents or gut samples; histology in some studies	Microbial richness may remain stable under some animal by-product replacement strategies; soybean-meal-type diets can induce stronger community rearrangement	Growth, intestinal and liver histology, or gut-health indicators were evaluated in some studies	Ingredient categories are heterogeneous; effects depend on inclusion level, antinutritional factors, background diet, and sampling design
Soybean meal or plant-protein replacement	Atlantic salmon; rainbow trout	Juvenile or seawater-stage salmonids; plant-based or soybean-meal challenge diets	Digesta, distal intestine, or gut samples	Shift toward lactic-acid-bacterial communities in some Atlantic salmon studies; altered diversity and dominant genera in rainbow trout	Soybean meal or saponin models can be associated with distal enteritis, epithelial stress, mucus-layer disturbance, or altered histology	Microbiota change is difficult to separate from antinutritional factors, undigested substrates, and inflammation; causal direction remains partly unresolved

Plant protein plus rapeseed oil or vegetable-oil replacement	Rainbow trout	Triploid rainbow trout under fishmeal or fish-oil replacement	Gut microbiome, including bacterial and fungal profiles	Bacterial and fungal α-diversity can decline; some genera become more dominant	Final body weight may remain unchanged despite microbial shifts	Unchanged growth does not prove preserved intestinal homeostasis; more barrier, immune, metabolite, and long-term phenotype data are needed
Insect meal, especially black soldier fly products	Atlantic salmon; rainbow trout	Seawater or grow-out Atlantic salmon; rainbow trout feeding trials	Digesta, mucosa, or intestinal samples	Enrichment of Lactobacillaceae, Actinomyces, chitinolytic Bacillaceae, Firmicutes, or Actinobacteria has been reported, depending on species and formulation	Some studies report no obvious adverse distal-intestinal histological or transcriptomic responses; growth and nutrient-utilization traits may also be assessed	Taxonomic enrichment should not be interpreted as beneficial by itself; effects depend on insect fraction, processing, inclusion level, basal diet, and sample type

Yeast or other single-cell proteins	Atlantic salmon; rainbow trout	Soybean-meal-based diets or plant-based diets; seawater stage in some Atlantic salmon studies	Digesta or intestinal samples	Yeast species and processing can alter soybean-meal-associated microbial trajectories; autolysed Cyberlindnera jadinii may enrich Pediococcus and predicted mucus O-glycan degradation pathways	Some studies include growth, health-related parameters, or predicted functional pathways	"Yeast protein" is not a single functional category; effects depend on yeast species, processing method, cell-wall composition, and background diet; predicted functions require validation
Fish-oil replacement with vegetable oil	Atlantic salmon	First-feeding Atlantic salmon	Gut microbiota samples	Complete replacement of fish oil with vegetable oil may not significantly alter gut microbiota composition at first feeding	Mainly microbiota response; host endpoints depend on study design	Developmental stage may override lipid-source effects; early-life findings may not extrapolate to later production stages

Partial replacement with algal oil, such as Schizochytrium oil	Atlantic salmon	Winter or seawater production context	Intestine, skin, and gill mucosal tissues	Microbiota effects are less directly resolved than mucosal-barrier responses	50% replacement can increase mucus-cell density, upregulate mucin and antimicrobial genes, and reduce HSP70 response; 100% replacement does not show the same advantage	Dose-dependent response; stronger evidence for mucosal-barrier modulation than for direct microbiome-mediated effects
Prebiotics	Atlantic salmon; rainbow trout	Adult Atlantic salmon or plant-based diet contexts in rainbow trout	Distal intestine, skin, intestinal contents, or gut-health samples	Oligosaccharide prebiotics can alter distal-intestinal and skin microbial communities; long-term responses may include reduced α-diversity, increased Mycoplasma, and decreased LAB in some Atlantic salmon studies	Histological and inflammatory indicators do not always improve; host metabolic responses may be detected in rainbow trout	Effects depend on host stage, basal diet, dose, duration, and sampling site; microbial shifts alone are insufficient to infer benefit

Probiotics, especially lactic-acid bacteria	Atlantic salmon; rainbow trout	Soybean-meal enteritis models, adult fish, or probiotic feeding trials	Digesta, mucus-layer communities, intestinal chyme, or distal intestine	Lactobacillus or other LAB may become dominant; network structures and community interactions can change	Some studies report altered metabolites, reduced lamina propria width, increased supranuclear vacuoles, or immune-gene modulation	Taxon enrichment alone does not prove improved health; effects depend on strain identity, dose, feeding rhythm, basal diet, and disease or enteritis context
Synbiotics or probiotic-prebiotic combinations	Mainly rainbow trout in current salmonid evidence	Controlled feeding or additive trials	Intestinal microbiome with metabolomic or multi-omics endpoints in some studies	Microbiota and metabolite profiles may shift together	Multi-omics studies can link microbial change to metabolic pathways and host growth or immune phenotypes	Evidence remains limited in number of studies; external validation across farms, diets, and life stages is still lacking

Postbiotics	Mainly rainbow trout	Feeding trials followed by bacterial challenge in some studies	Intestinal microbiota or bacterial count data	Increased microbial diversity or lactic-acid-bacterial counts have been reported	Improved resistance to Lactococcus garvieae or improved survival after Yersinia ruckeri challenge has been reported in challenge tests	Evidence is concentrated in rainbow trout; direct evidence for Atlantic salmon postbiotics remains scarce; mechanisms require stronger validation
